# Biodegradation of Amphipathic Fluorinated Peptides Reveals a New Bacterial Defluorinating Activity and a New Source of Natural Organofluorine Compounds

**DOI:** 10.1021/acs.est.3c01240

**Published:** 2023-06-21

**Authors:** Mohd Faheem Khan, Suvrat Chowdhary, Beate Koksch, Cormac D. Murphy

**Affiliations:** † School of Biomolecular and Biomedical Science, University College Dublin, Belfield, Dublin 4, Ireland; ‡ Institute of Chemistry and Biochemistry, Freie Universität Berlin, Arnimallee 20, Berlin 14195, Germany

**Keywords:** amino acid, dehalogenase, fluoroacetate, protease, organofluorine

## Abstract

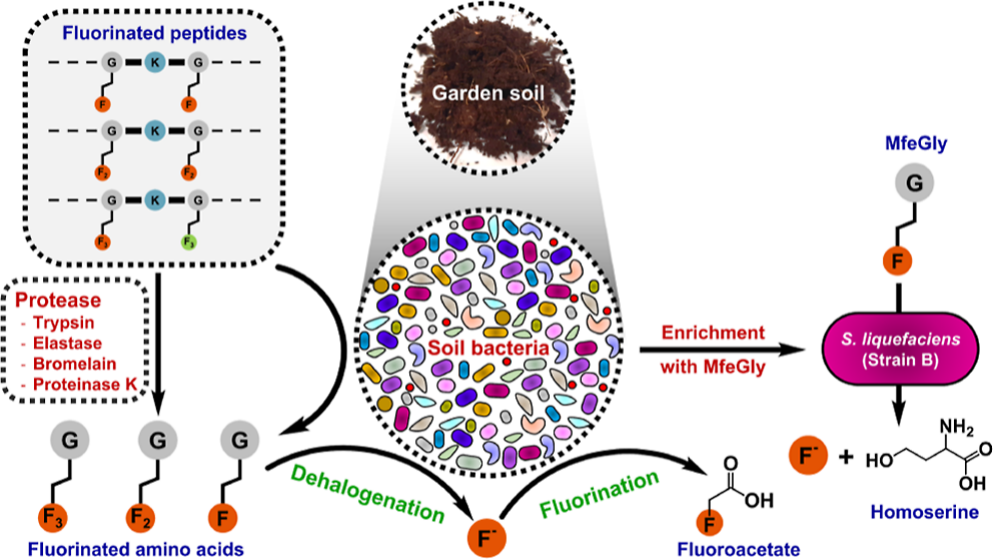

Three peptides comprising mono-, di-, and tri-fluoroethylglycine (MfeGly, DfeGly, and TfeGly) residues alternating with lysine were digested by readily available proteases (elastase, bromelain, trypsin, and proteinase K). The degree of degradation depended on the enzyme employed and the extent of fluorination. Incubation of the peptides with a microbial consortium from garden soil resulted in degradation, yielding fluoride ions. Further biodegradation studies conducted with the individual fluorinated amino acids demonstrated that the degree of defluorination followed the sequence MfeGly > DfeGly > TfeGly. Enrichment of the soil bacteria employing MfeGly as a sole carbon and energy source resulted in the isolation of a bacterium, which was identified as *Serratia liquefaciens*. Cell-free extracts of this bacterium enzymatically defluorinated MfeGly, yielding fluoride ion and homoserine. *In silico* analysis of the genome revealed the presence of a gene that putatively codes for a dehalogenase. However, the low overall homology to known enzymes suggests a potentially new hydrolase that can degrade monofluorinated compounds. ^19^F NMR analysis of aqueous soil extracts revealed the unexpected presence of trifluoroacetate, fluoride ion, and fluoroacetate. Growth of the soil consortium in tryptone soya broth supplemented with fluoride ions resulted in fluoroacetate production; thus, bacteria in the soil produce and degrade organofluorine compounds.

## Abstract 2

New fluorinated compounds should be assessed for their biodegradability. In this paper, the biodegradation of novel fluorinated peptides was investigated, leading to unexpected activities being detected in soil bacteria.

## 1 Introduction

Fluorinated organic compounds are used in numerous applications including pharmaceuticals (∼20% are fluorinated), agrochemicals (∼30% are fluorinated), aerosols, refrigerants, degreasers, fire-fighting foams, and stain-resistant materials.^1^ Their widespread use has resulted in these compounds polluting the environment, where they can have significant impacts on wild-life and human health.^2^ For example, the fluorinated antidepressant fluoxetine is present in aquatic environments and has measurable physiological impacts on fish,^3^ and the effects of per- and poly-fluorinated substances on human health are of immediate concern.^4,5^ Therefore, it is important that the potential environmental fate of organofluorine compounds is evaluated at an early stage, so that any potential hazards can be avoided.^6^ Furthermore, an understanding of how different fluorinated moieties are biodegraded enables the development of new compounds that are “benign by design”.

Fluorinated compounds can be slow to degrade in the environment owing to the stability of the carbon–fluorine bond and the lack of enzymes that have evolved to specifically cleave this bond.^7^ Microorganisms can degrade fluorinated compounds along common catabolic pathways, resulting in either the production of dead-end fluorometabolites or the spontaneous release of fluoride ions from an unstable intermediate.^8^ Thus, microorganisms might be applied to the bioremediation of fluorinated pollutants; for example, fluoxetine, which is not fully removed from wastewater treatment plants, can be degraded by some microorganisms.^9^ Khan and Murphy^10^ reported that environmental bacteria, such as *Bacillus* and *Pseudomonas*, could use fluoxetine as a sole carbon and energy source, producing 4-(trifluoromethyl)phenol as a fluorometabolite, which was further catabolized *via* meta-cleavage to semialdehyde intermediates that were light sensitive and spontaneously degraded releasing fluoride ions.

Self-assembling peptides (SAPs), which are composed of alternating hydrophobic/hydrophilic amino acid residues, are of interest in applications in tissue engineering owing to their biocompatibility, ease of biodegradation, and biofunctionality.^11,12^ Inclusion of fluorinated amino acids can fine tune the biophysical properties of amphipathic peptide hydrophobicity, peptide secondary structure formation/protein folding, and self-assembly, often with favorable outcomes.^13−17^ However, most studies focused on the single and site-specific replacement of canonical amino acids with their fluorinated counterparts, whereas the chemical and biological nature of synthetic peptides containing a large proportion of fluorinated amino acids remains largely unexplored. We recently designed a small library of cationic (Lys-rich) and polyfluorinated SAPs including the iterative incorporation of either α-aminobutyric acid (Abu), (2*S*)-4-monofluoroethylglycine (MfeGly), (2*S*)-4,4-difluoroethylglycine (DfeGly), and (2*S*)-4,4,4-trifluoroethylglycine (TfeGly) as hydrophobic components. Fluorine-specific interactions were found to beneficially impart peptide folding up to the fabrication of physiological peptide hydrogels equipped with fluorination-enhanced mechanical stiffness.^18^ Incorporation of fluorine-containing amino acids into peptides is highly likely to be a feature of future novel biomaterials; thus, how these peptides are biodegraded is important from environmental and health perspectives. In this paper, we describe the enzymatic degradation of three fluorinated peptides, MfeGlyK16, DfeGlyK16, and TfeGlyK16, and explore in depth the microbial degradation of the fluorinated amino acids.

## 2 Materials and Methods

### 2.1 General Methods and Chemicals

The SAPs were prepared according to standard microwave-assisted SPPS and purification protocols recently published by Koksch and co-workers.^18^ The synthesis of the fluorinated amino acids MfeGly, DfeGly, and TfeGly is described in the Supporting Information, plus the relevant NMR spectra (Figures S1–S6). High-resolution mass spectrometry (HRMS) spectra were recorded on an Agilent 6220 ESI-TOF MS instrument (Agilent Technologies, Santa Clara, CA, USA), and MassHunter Workstation software version B.02.00 (Agilent Technologies, Santa Clara, CA, USA) was used for data analysis. Unless otherwise stated, all other compounds were obtained from Merck (Arklow, Ireland). The API 20E strip was obtained from Biomerieux. The enzymes β-trypsin (pancreas), elastase (pancreas), proteinase K (*Tritirachium album*), and bromelain (pineapple) were obtained from Sigma-Aldrich (Steinheim, Germany).

### 2.2 Peptide Digestion Assay

Freeze-dried peptides AbuK16, MfeGlyK16, DfeGlyK16, and TfeGlyK16 were dissolved in 100 μL of buffer (50 mM bis-tris propane + 20 mM CaCl_2_, pH 8) to a final concentration of 500 μM. The peptides were incubated with different proteolytic enzymes (10 μL in buffer): trypsin (0.075 μM), elastase (0.45 μM), proteinase K (0.075 μM), and bromelain (4.5 μM). The experiments were incubated at either 37 °C (trypsin and proteinase K) or 30 °C (elastase and bromelain) in a Thriller thermoshaker-incubator (VWR International, Radnor, PA, USA) with shaking at 300 rpm. Aliquots (15 μL) were taken at fixed time points and quenched with 90 μL of a solution of 30% acetic acid in water containing 133 μM Ac-[4]Abz-Gly-OH as a reference. Peptide degradation was monitored by high-performance liquid chromatography (HPLC) using a Hitachi Primaide UV-HPLC system (VWR/Hitachi, Darmstadt, Germany). A Kinetex RP-C18 (5 μM, 100 Å, 250 × 4.6 mm, Phenomenex, USA) column and a SecurityGuard Cartridge Kit equipped with a C18 cartridge (4 × 3.0 mm, Phenomenex, USA) as a pre-column was used. The mobile phases employed were 0.1% aqueous trifluoroacetyl (TFA) (solvent A) and acetonitrile + 0.1% TFA (solvent B). The solvent conditions for analysis were as follows: 0–5 min, 5% B; 5–20 min, 5–90% B; 20–23 min, 90–100% B; 23–25 min, 100% B; 25–27 min, 100–5% B; and 27–30 min, 5% B. In all cases, the peaks corresponding to each peptide sample (full-length peptides) and the reference sample were integrated and used to determine the amount of the substrate still present in solution. The content of starting material after 5 min was termed as absolute amount for simplicity. All experiments were performed in triplicates, and error bars were obtained as standard deviations of measurements. Control experiments in which the SAPs were incubated in the absence of enzyme were conducted, which showed that they were stable over 48 h (Figure S8).

### 2.3 Microorganisms

The soil microbial consortium (SM) was obtained by inoculating tryptone soya broth (TSB, 50 mL) with approx. 1 g of garden soil collected in Dublin, Ireland (GPS coordinates 53°16′50.0″N 6°16′05.0″W) and incubating at 30 °C for 48 h and 200 rpm. Uncultivated soil was also collected from two locations in Ireland (53°11′59.7″N 6°12′50.8″W and 53°15′39.9″N 6°15′31.1″W), and the microbes were cultivated in the same way. These cultures were labeled SM1 and SM2, respectively. The culture was diluted (×5) in fresh TSB containing 15% glycerol, and aliquots (1 mL) were stored at −80 °C and used as inocula for the different experiments. For biodegradation experiments, the inoculum (200 μL) was added to TSB (20 mL) and incubated for 24 h at 30 °C and 200 rpm. The fluorinated substrate (2 mg) was added directly to the cultures, which were incubated for 48 h; for resting cell experiments, the cells were harvested after 24 h growth, washed with *N*-(2-hydroxyethyl)piperazine-*N*′-ethanesulfonic acid (HEPES) buffer, and resuspended in the same buffer to the original volume. The fluorinated substrate was added and incubated for 48 h. The supernatants from whole cell experiments were recovered by centrifugation, extracted with ethyl acetate, and analyzed for fluorometabolite production.

*Caballeronia* sp. DSM 8341 was obtained from DSMZ-German Collection of Microorganisms and Cell Cultures (Leibniz Institute, GmbH) and maintained on tryptone soya agar. For experiments assessing defluorination, the bacterium was initially cultivated at 30 °C in liquid culture on TSB (10 mL) supplemented with fluoroacetate (1 mg) or MfeGly (1 mg) for 48 h. The cells were harvested by centrifugation, washed with HEPES buffer (pH 7), and resuspended in the same buffer (1 mL). The cell suspension was incubated with the fluorinated substrate (0.2 mg) for 6 h at 30 °C with shaking at 300 rpm using an Eppendorf Thermomixer C dry block heater. The supernatant was recovered by centrifugation, and the fluoride ion was measured as described below.

### 2.4 Isolation of MfeGly-Degrading Strains

Molten agar (2%) supplemented with 20 mM filter-sterilized MfeGly was poured onto a Petri dish, inoculated with the soil microbe inoculum (100 μL), and incubated at 30 °C for 48 h. The grown colonies (10) were re-streaked onto separate TSA plates and incubated at 30 °C for 24 h. The colonies were screened for production of homoserine and fluoride release from biotransformation of MfeGly. One colony was selected (named strain B) based on above assay and sent to MicrobesNG (Birmingham, UK) for whole genome sequencing. The bacterium was identified from its 16S rRNA gene sequence and with an API (analytical profile index) strip. A phylogenetic tree was constructed using similar 16S rRNA sequences collected using nucleotide BLAST analysis and aligning them using the Kalign algorithm. The phylogenetic tree data were collected from Kalign and visualized using iTOL v6 online tool (https://itol.embl.de/). The genome was searched for a gene coding for fluoroacetate dehalogenase using the tblastn function of NCBI, with a proposed fluoroacetate dehalogenase from *Serratia marcescens* as the query sequence (accession number CVE64293.1).

### 2.5 Measuring Defluorinating Activity in Cell-Free Extracts

The SM or the isolated strain was grown in TSB (20 mL) as described. Cells were harvested by centrifugation, and the supernatants were discarded. The cell pellets were resuspended in 20 mM HEPES buffer (pH 7.0) and lysed by sonication (Sonics 130 W ultrasonic processor) at 30% amplitude, 10 s pulse on, and 15 s pulse off for a total of 5 min, on ice. The cell lysates were centrifuged at 16,000 rpm, 4 °C for 20 min, and the supernatants were collected and used as cell-free extracts. Defluorinating activity was measured by adding 0.2 mg of the fluorinated substrate to 1 mL of the cell-free extract, incubating for 6 h and measuring fluoride ions.

### 2.6 Analysis of Fluorinated Metabolites

The freeze-dried supernatant was redissolved in D_2_O, and samples were analyzed by ^19^F NMR using a Varian 400 MHz spectrometer. Gas chromatography–mass spectrometry (GC–MS) analysis was carried out according to the method described by Khan and Murphy.^10^ Briefly, the ethyl acetate culture extracts were dried under N_2_ gas and derivatized by silylation using 40 μL of *N*-methyl-*N*-(trimethylsilyl)trifluoroacetamide at 100 °C for 45 min. The final volume was adjusted to 500 μL by adding ethyl acetate. The samples were analyzed using a 7890B N Agilent GC system equipped with a HP-5MS capillary column (30 m × 0.25 mm × 0.33 μm) and a 5977 A mass-selective detector. The split mode (20:1) was used for the sample injection (1 μL) onto the column. The oven temperature was initially set at 90 °C for 3 min and then raised to 300 °C at 10 °C/min rate. The mass-selective detector was operated in the scan mode.

Rapid evaluation of fluoride ion liberation in the resting cell and cell-free experiments was initially determined using a colorimetric assay developed by Bygd *et al.*,^19^ which employs a lanthanum–alizarin complex that turns purple in the presence of fluoride ions. To the wells of a 96-well plate were added 20 μL of alizarin-3-methyliminodiacetic acid (500 μM), 20 μL of lanthanum(III) nitrate hexahydrate (500 μM), 50 μL of acetone, 10 μL of acetate buffer (1.68 M), and 100 μL of sample or NaF standard. All stock solutions were prepared in 20 mM HEPES buffer (pH 7.0). The mixture was allowed to stand for 10 min at room temperature, and the absorbance was measured at 620 and 530 nm using an Epoch 2 microplate spectrophotometer (Biotek Instrumentations). The ratio of the absorbance measurements was used to indicate fluoride ion release. The concentration of fluoride ions was subsequently measured using a combination F^–^ ion-selective electrode (Thermo Orion) using the previously described method.^10^

## 3 Results and Discussion

### 3.1 Fluorinated Amphipathic Peptides Are Proteolytically Degraded

**Figure 1:**
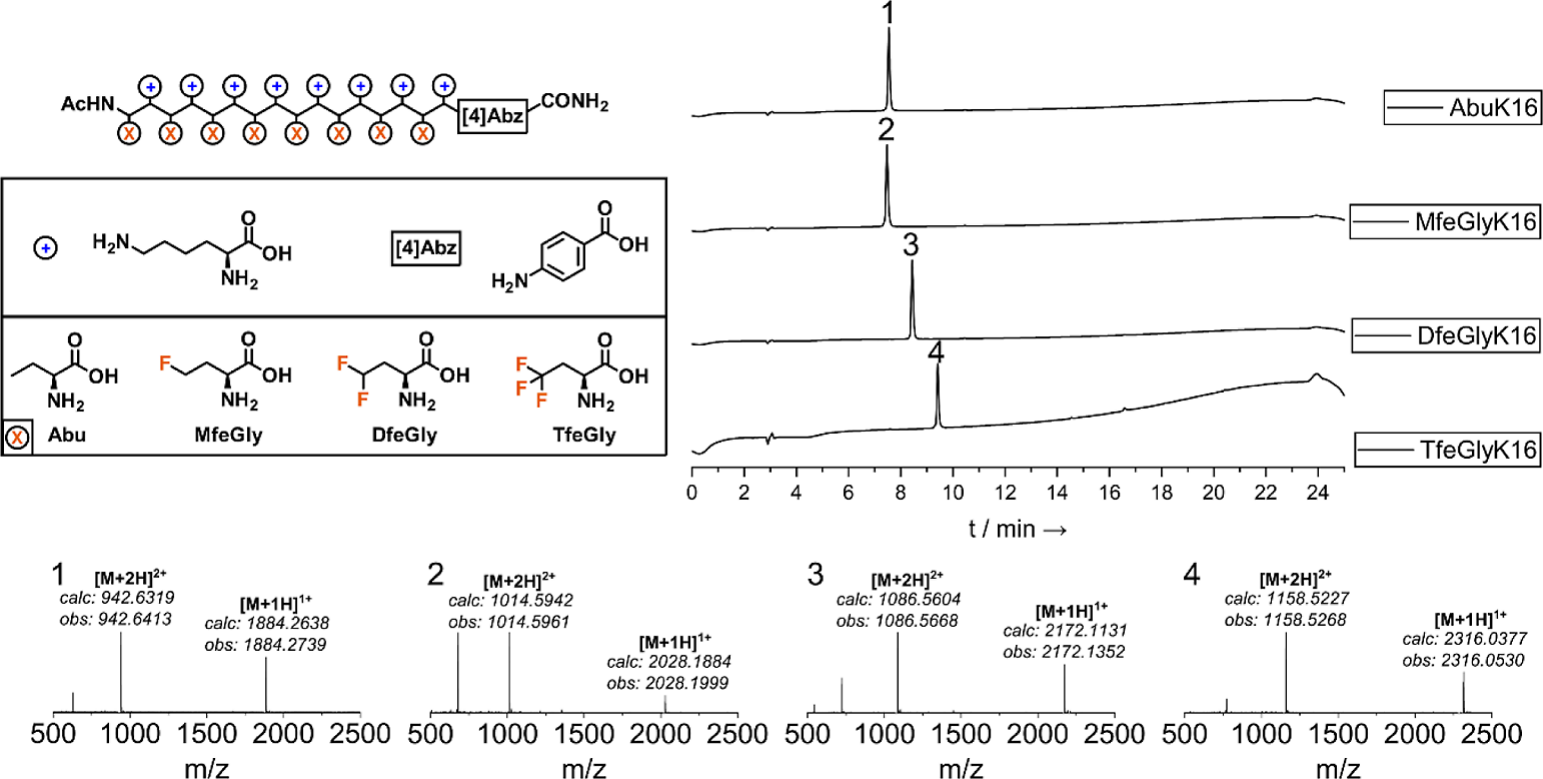
Analysis of (fluorinated) peptides AbuK16, MfeGlyK16, DfeGlyK16, and TfeGlyK16. Successful synthesis (SPPS) and purification (HPLC and HRMS) was confirmed *via* HPLC chromatograms and ESI–ToF mass spectrometry. HPLC conditions: (A) H_2_O + 0.1% TFA/(B) ACN + 0.1% TFA with a gradient of 10–80% (B) over 18 min.

The increasing use of fluorinated compounds in the range of applications has environmental implications, owing to the stability of the carbon–fluorine bond.^20^ In prior work, we examined the SAP AbuK16 (Ac-(Abu-Lys)_8_-[4]Abz-NH_2_), MfeGlyK16 (Ac-(MfeGly-Lys)_8_-[4]Abz-NH_2_), DfeGlyK16 (Ac-(DfeGly-Lys)_8_-[4]Abz-NH_2_), and TfeGlyK16 (Ac-(TfeGly-Lys)_8_-[4]Abz-NH_2_) (Figure 1) in the context of secondary structure formation, supramolecular self-assembly, and hydrogel formation.^18^ The influence of fluorine-containing amino acids on the enzymatic resistance of peptides has been investigated by the research groups of Marsh, Kumar, and Koksch.^21−26^ Several parameters were identified to significantly affect proteolytic stability like the amino acids’ spatial demand, hydrophobicity, residual position, and electrostatic attractions. However, the resulting effects on enzyme–substrate recognition are not predictable.^27−29^

**Figure 2:**
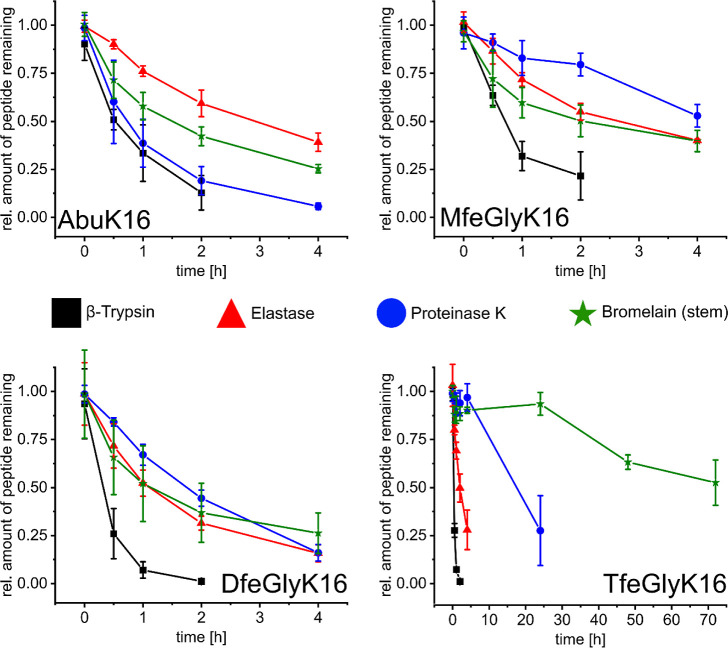
Proteolytic digestion of AbuK16, MfeGlyK16, DfeGlyK16, and TfeGlyK16 by the serine proteases β-trypsin, elastase, proteinase K, and bromelain (pineapple stem) in 50 mM bis-tris propane + 20 mM CaCl_2_, pH 8, at 37 °C (β-trypsin and proteinase K) or 30 °C (elastase and bromelain). Real-time detection of peptide proteolysis and calculation of remaining peptide amounts were accomplished by HPLC analysis (DAD-280 nm).

In the present work, we selected four different enzymes (β-trypsin, elastase, proteinase K, and bromelain) to investigate the proteolytic stability of our peptides. These enzymes were employed as model proteases to assess the general proteolytic stability of the fluorinated peptides. The enzyme β-trypsin strongly prefers to cleave the amide bonds of cationic P1 residues such as Arg or Lys while elastase shows a marked S1 specificity for short aliphatic P1 residues.^30,31^ The activity of proteinase K is rather unspecific but was described in earlier reports to own a preference toward aromatic and hydrophobic amino acids.^32,33^ A well-known cysteine protease is bromelain derived from pineapple stem extracts. This member of the papain family strongly favors basic amino acids similar to β-trypsin.^34^ For real-time detection of peptide proteolysis, HPLC analysis of quenched aliquots was employed, and non-fluorinated AbuK16 was set as an internal standard. The digestion plots are presented in Figure 2.

In the case of β-trypsin, we observed a decrease in proteolytic resistance with peptides having a higher degree of fluorination (DfeGly and TfeGly). After 2 h, both peptides DfeGlyK16 and TfeGlyK16 were mostly consumed, and comparably higher amounts of peptide were detected for AbuK16 and MfeGlyK16. The products of these digestions were analyzed by HPLC, and the expected dipeptides Abu–Lys, MfeGly–Lys, DfeGly–Lys, and TfeGly–Lys were detected (Figure S9). For elastase, similar findings were observable after 4 h of incubation, with lower amounts of DfeGlyK16 and TfeGlyK16 detected than AbuK16 and MfeGlyK16. These conclusions, however, cannot be generalized since proteinase K and bromelain reveal different trends. After 4 h of incubation, the digestion plots of proteinase K display a higher proteolytic stability of MfeGlyK16 than that for AbuK16 and DfeGlyK16. Ultimately, the most fluorinated peptide TfeGlyK16 possesses a pronounced stability against proteinase K after 4 h incubation, but the peptide was largely digested within 24 h. The TfeGly side chain also provides the best protection of this SAP scaffold toward the cysteine protease bromelain. The peptides AbuK16, MfeGlyK16, and DfeGlyK16 were digested, whereas TfeGlyK16 was found to be resistant to this protease; even after 3 days incubation, noticeable amounts of this peptide were still present.

Thus, the fluorinated SAPs are broadly susceptible to proteolytic degradation, and these experiments provided some indication of the potential fate of the compounds in the environment. However, as there are many other enzyme activities that are present in the biosphere, a more detailed investigation of the peptides’ degradation was warranted.

### 3.2 Fluorinated Peptides Are Microbially Degraded Yielding Fluoride Ions

**Figure 3:**
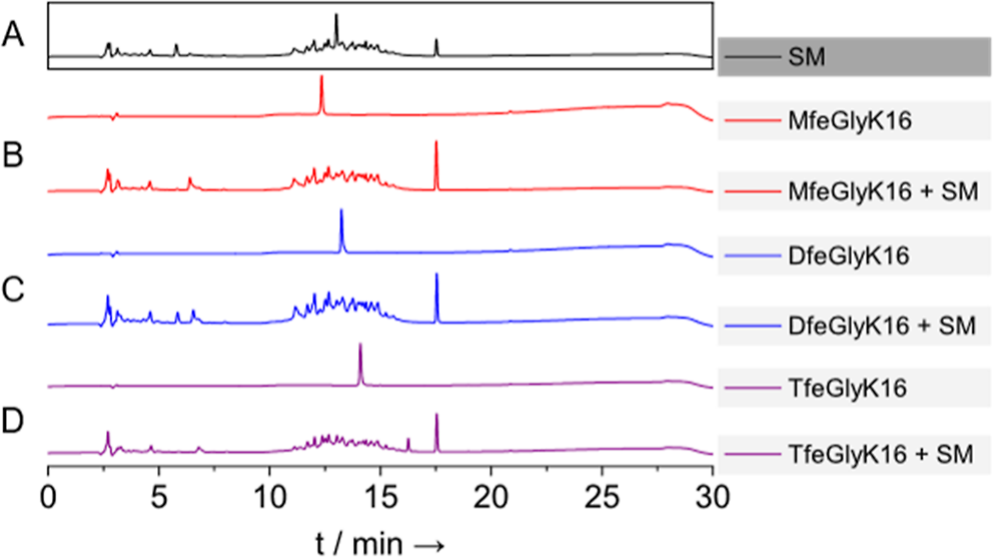
HPLC chromatograms of the soil microbe extract after incubation with the fluorinated peptides. (A) Chromatogram of the soil microbes without any peptide addition; (B–D) chromatograms of MfeGlyK16, DfeGlyK16, and TfeGlyK16 alone and after incubation with the soil microbes.

Further biodegradation studies on the peptides were conducted by incubating them with a microbial community (SM) obtained from a garden soil, which is likely to have a broad range of proteases and other enzymes that might contribute to the biotransformation of the substrates. The consortium was initially cultivated for 24 h before the peptides were added to the growing cells, and the incubation continued for 48 h. The degradation was evaluated by HPLC, showing disappearance of the peptide from culture supernatants (Figure 3), and ^19^F NMR, which confirmed the disappearance of the peptides and revealed the appearance of new resonances, including that of fluoride ions at approximately δ −122 ppm (Figure S10). These data demonstrated that the microorganisms in the soil extensively degraded all of the peptides, yielding new fluorinated metabolites (from TfeGlyK16) and fluoride ions.

**Figure 4:**
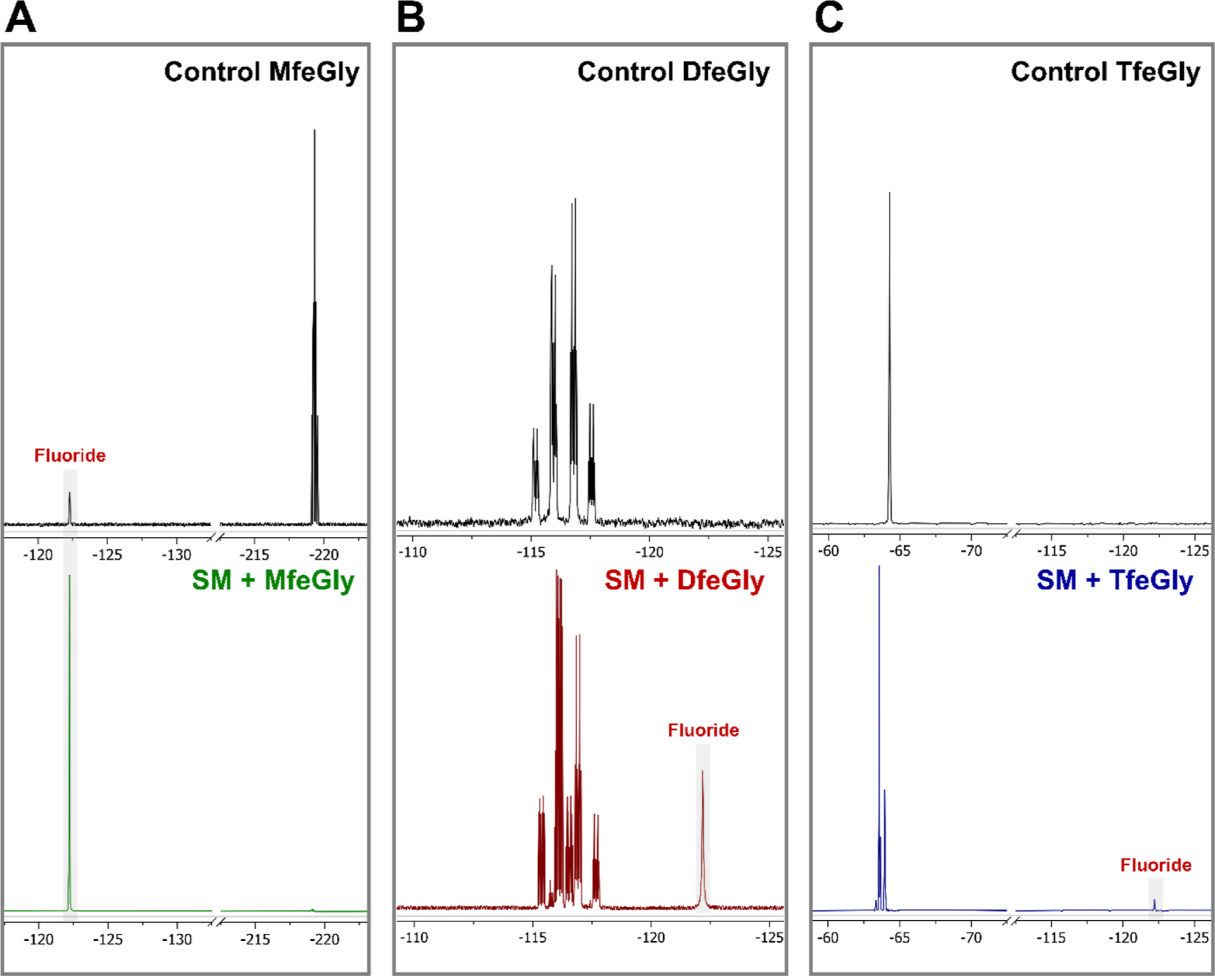
^19^F NMR spectra of supernatants of resting cells of soil microbes incubated with the fluorinated amino acids. In the control experiments, the amino acids were incubated with HEPES buffer for 48 h.

It is most likely that the degradation by the soil microbes is initiated by peptide hydrolysis to the individual amino acids. To investigate if there was further degradation of the different fluorinated amino acids, they were incubated with growing and resting cells of soil bacteria, and the products were monitored by ^19^F NMR (Figure 4). Through comparison of the relative resonance heights, the degree of amino acid defluorination decreased as the number of fluorine atoms increased (MfeGly > DfeGly > TfeGly). MfeGly was completely degraded by soil microbes as only the resonance for fluoride ions was observed. The degradation of DfeGly and TfeGly is less straightforward, and there appears to be other fluorometabolites produced. In the case of DfeGly, the signal around δ −117 ppm was more complicated after incubation with the soil microbes indicating the presence of another fluorinated compound, and the TfeGly resonance (δ −64 ppm) shifts upfield. These observations indicate a change to the amino acids away from the site of fluorination, such as deamination or decarboxylation. Fluorinated amino acids are known to be biotransformed by enzymes associated with amino acid catabolism. For example, it was reported that incubation of 4,4,4-trifluoro-dl-valine with a *Bacillus* sp. resulted in the production of 4,4,4-trifluoro-2-hydroxy-3-methylbutanoic acid.^35^ Biodefluorination of fluorinated amino acids is not well known; one example was reported by Donnelly and Murphy^36^ who observed defluorination of 4-fluoroglutamic acid, which was accompanied by ammonia release. Although the enzymatic mechanism was not revealed, it is likely that the deamination of the amino acid leads to non-enzymatic fluoride release, which is supported by more recent work by Wu and Deng^37^ who demonstrated that 4-fluorothreonine was defluorinated by threonine deaminase.

### 3.3 Soil Bacteria Have Dehalogenase Activity

**Figure 5:**
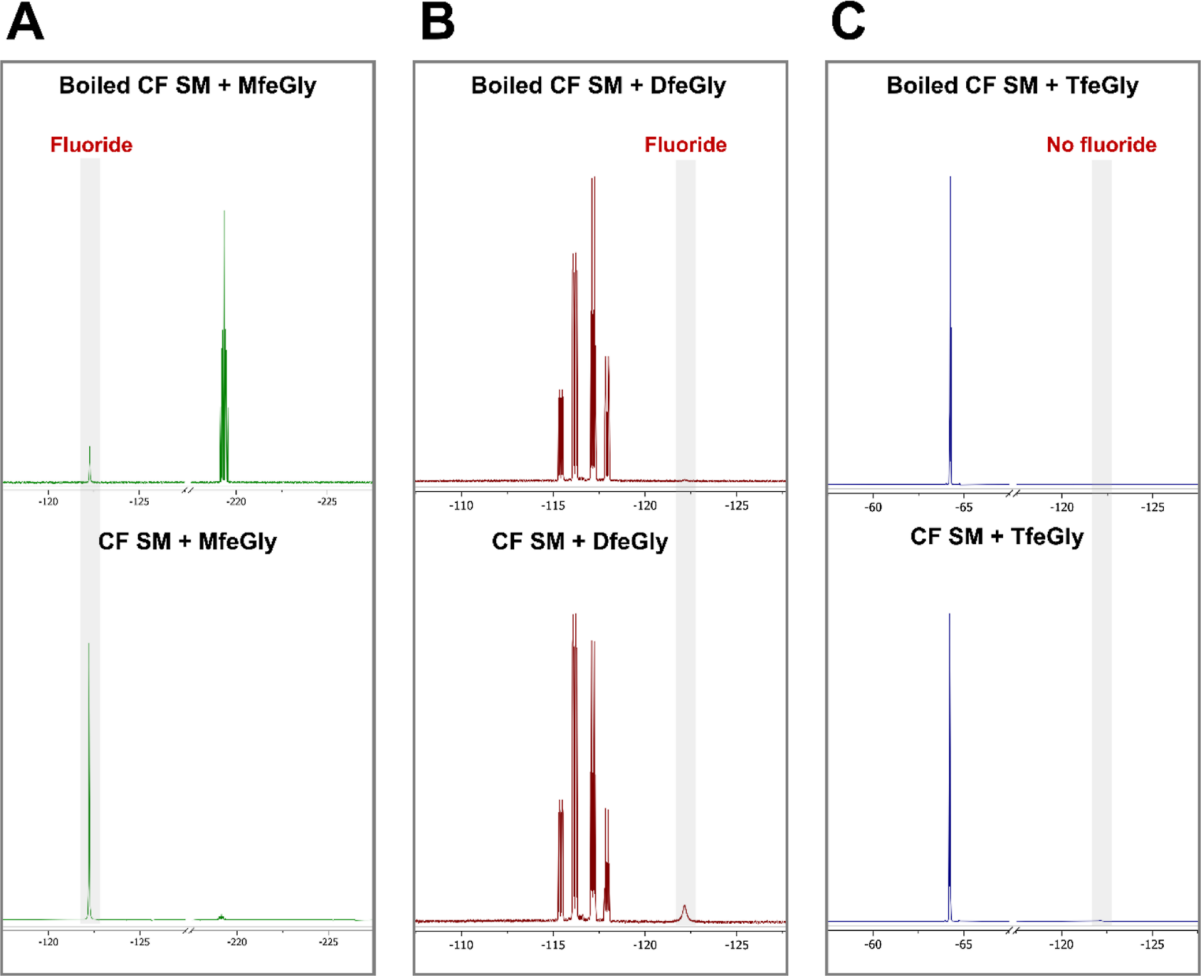
^19^F NMR of the cell-free extracts (CF) from the SM incubated with fluorinated amino acids. Incubation of the amino acids with boiled CF acted as controls.

Biological defluorination can occur *via* specific enzymatic dehalogenation or through catabolism of a fluorinated compound to an unstable intermediate that spontaneously eliminates fluoride ions.^38^ There is only one class of microbial enzyme, fluoroacetate dehalogenase, known to catalyze specific carbon–fluorine cleavage. This enzyme catalyzes the hydrolysis of fluoroacetate, yielding fluoride ion and glycolate, and there are some bacteria known to possess the enzyme.^39−41^ To investigate the mechanism of defluorination of the fluorinated ethylglycines, the amino acids were incubated with a cell-free extract of the microbial consortium. Fluoride ions were readily detected by ^19^F NMR in extracts incubated with MfeGly for 6 h (Figure 5), whereas only a very small fluoride ion resonance was detected in control experiments employing a boiled cell extract. Most DfeGly was untransformed, but a small fluoride signal was observed in the spectrum recorded; TfeGly was not defluorinated by the cell extract.

**Figure 6:**
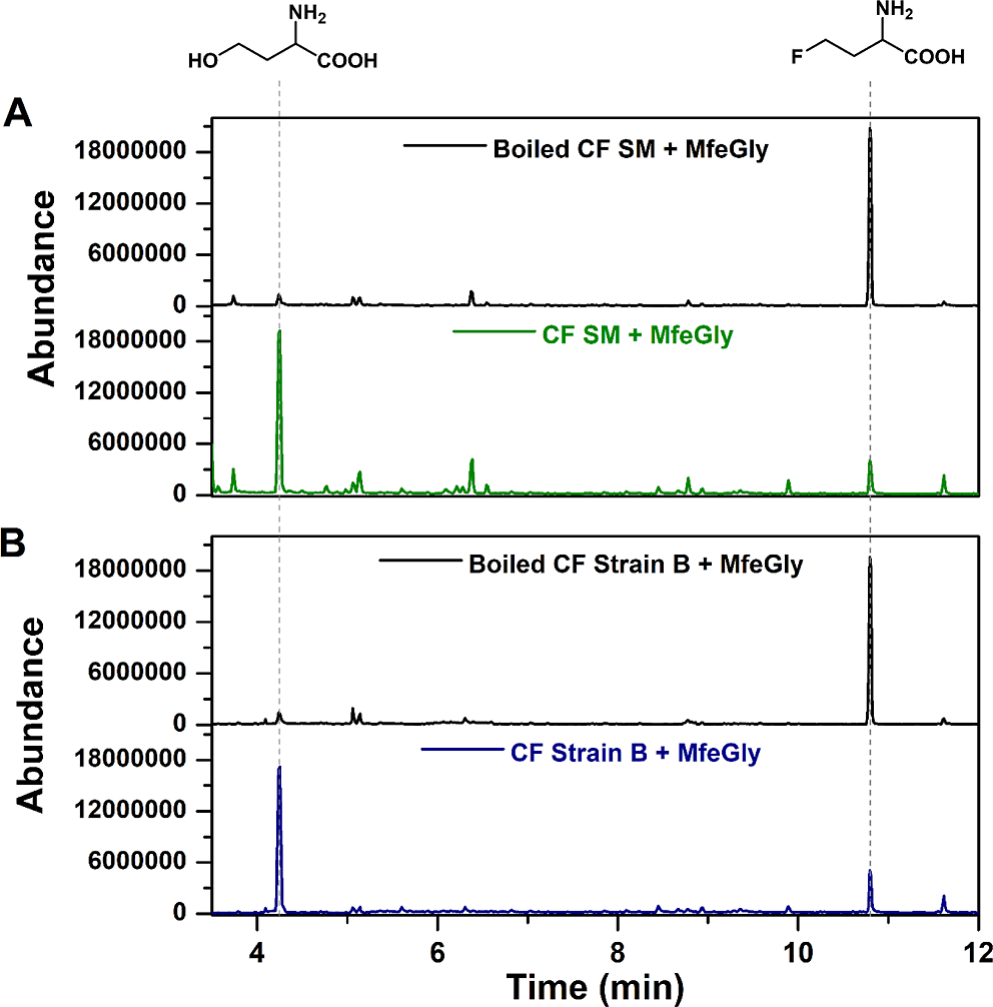
Total Ion Chromatograms (TIC) of silylated CF extracts from soil microbes (A) and strain B (B) after incubation with MfeGly showing the increase in the peak at 4.4 min compared with that of a control experiment with boiled extract. The mass spectra of the substrate and product are given in Figure S11.

Since the MfeGly was the best substrate for defluorination, the possible product of the reaction was sought using GC–MS, following silylation of a sample of the assay. Figure 6A shows chromatograms of the compounds detected after incubation of MfeGly with active and boiled cell-free extract. The peak for MfeGly elutes after 11 min and is detected in both assays, but the peak is much diminished in the active extract relative to the other peaks present. Also apparent in the chromatograms is a peak at 4.3 min, which is also present in both experiments, but its relative height is noticeably greater in the active extract. The mass spectrum of this peak matches that of homoserine, and it is most likely that this is the immediate product of defluorination of MfeGly. The implication of this observation is that there are microorganisms in the soil consortium that have a specific defluorinating enzyme that hydrolytically cleaves the carbon–fluorine bond of MfeGly. Also, since there was little or no fluoride release from DfeGly and TfeGly in cell-free experiments, a different mechanism must be responsible for the fluoride release that was observed in experiments with whole cell cultures. There are several reasons why defluorinating activity toward these substrates was not detected, for example, the enzyme(s) required might be membrane-associated and so would not be active in the cell-free extract, or a necessary cofactor is missing, or the lysis process may have caused denaturation.

### 3.4 Some Bacteria Can Employ MfeGly as a Sole Source of Carbon and Energy

To further investigate the enzymatic defluorination of MfeGly, an enrichment culture was established in which mineral medium containing the amino acid as a sole source of carbon and energy was inoculated with the soil consortium. After 48 h, turbidity was observed indicating microbial growth, and the liquid culture was used to streak agar plates. Several different colonies were apparent on the plates, indicating that more than one microorganism was enriched. One of these colonies, designated strain B, was isolated *via* streaking and separately assessed for its defluorinating properties. The strain was initially cultivated on a rich medium (TSB), and cell-free extracts were prepared, which were incubated with MfeGly. Fluoride ions were colorimetrically detected, and the increase in homoserine observed by GC–MS (Figure 6B) showed that the bacterium could degrade the substrate in the same manner as the cell extract prepared from the mixed community of soil microbes.

**Figure 7:**
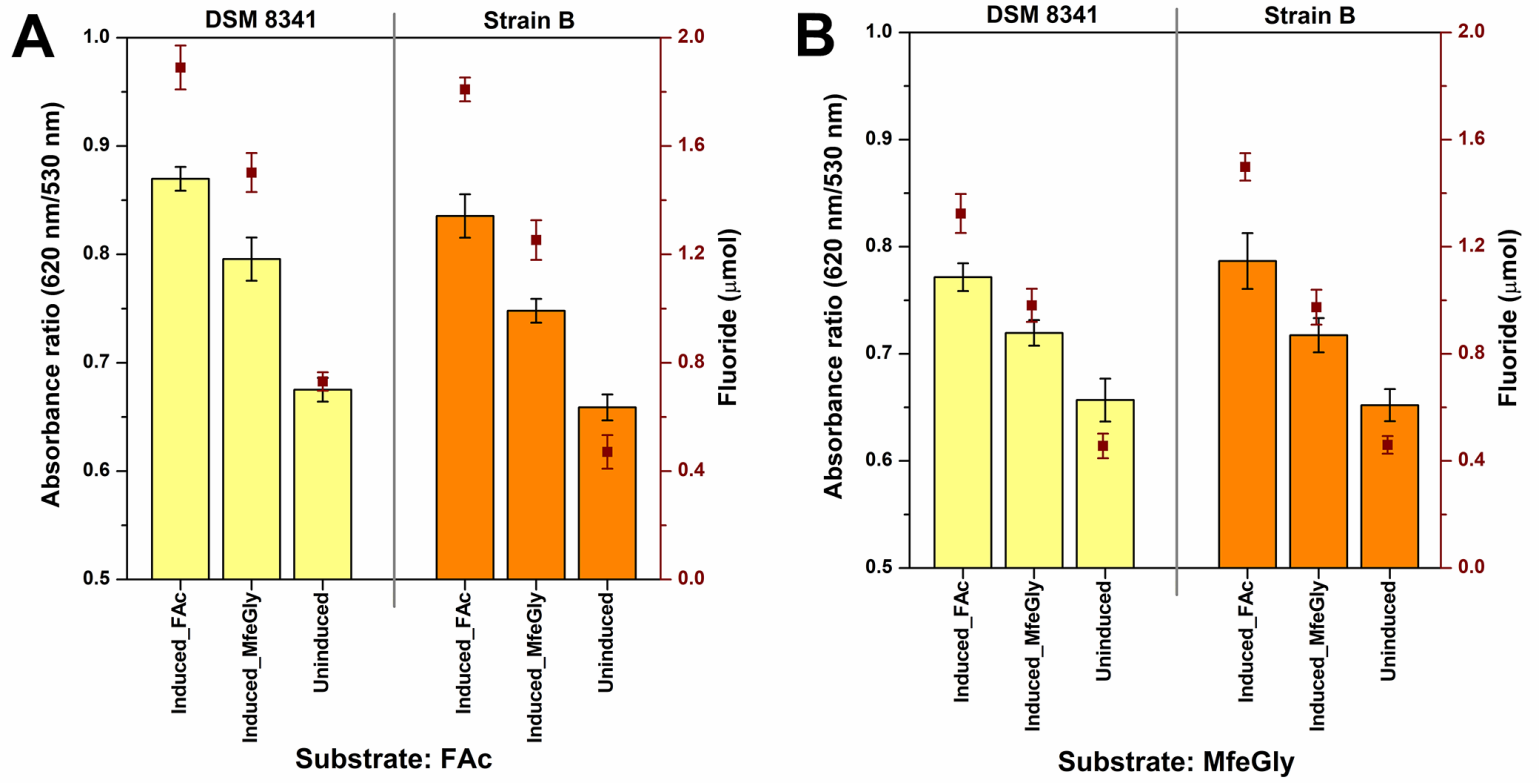
Colorimetric detection of fluoride ions after DSM8341 and strain B were incubated with either fluoroacetate or MfeGly for 6 h. The bacteria were grown in TSB with (induced) and without (uninduced) added fluorinated substrates. The histogram shows the ratio of 620:530 nm absorbance, and a ratio 0.6 indicates the presence of fluoride ions. The red squares indicate fluoride ion measured using an ion-selective electrode. A negative control experiment was conducted with *Escherichia coli*, which is not known to degrade fluorinated compounds, and no fluoride ion was detected using the colorimetric method.

There are no reports in the literature on the enzyme-catalyzed defluorination of MfeGly, so it was possible that this was a novel activity; however, we were also cognizant that the only known microbial defluorinase, fluoroacetate dehalogenase, had never been tested with this substrate. Therefore, an experiment was conducted in which the isolate was assessed for its ability to degrade fluoroacetate, and a known fluoroacetate-degrading bacterium, *Caballeronia* sp. DSM 8341 (previously *Pseudomonas fluorescens*), was investigated for its ability to degrade MfeGly. The bacteria were grown in TSB in the presence and absence of either fluoroacetate (FAc) or MfeGly, to induce defluorinase activity. The cells were harvested, resuspended in buffer, and incubated with the fluorinated substrates. Fluoride ions were measured colorimetrically and with an ion-selective electrode (Figure 7), which showed that both bacteria could defluorinate both substrates, strongly suggesting that the enzyme present in strain B was related to the known fluoroacetate dehalogenases.

**Figure 8:**
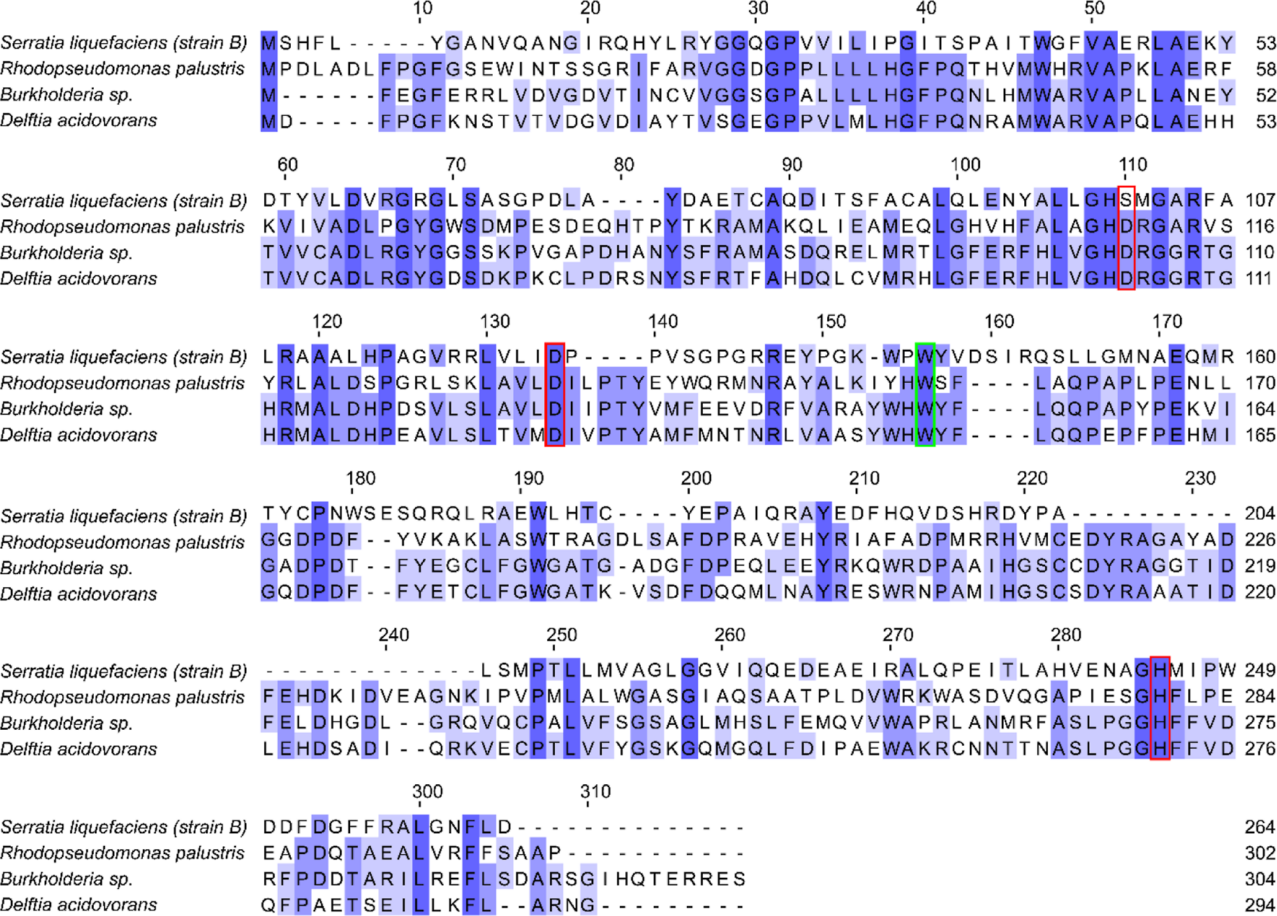
Alignment of the protein identified from strain B as a possible fluoroacetate dehalogenase and the known fluoroacetate dehalogenases from *Rhodopseudomonas palustris* (protein ID Q6NAM1), *Burkholderia* sp. (protein ID Q1JU72), and *Delftia acidovorans* (protein ID Q01398). The residues in the red boxes are the catalytic triad in the active site; the tryptophan residue (green box) was previously identified as being essential for defluorination of fluoroacetate.^42^

The genome of strain B was sequenced, and its 16S rRNA gene sequence indicated that it was most closely related to *Serratia liquefaciens* (Figure S12), which was confirmed using an API 20E strip. A search of the NCBI database revealed a gene putatively labeled as fluoroacetate dehalogenase from the related species *S. marcescens* (accession number CVE64293.1). This sequence was used as the subject sequence to search strain B’s genome for similar genes using translated BLAST (tBlastn). One gene was identified that had a translated sequence with 81% identity to the *S. marcescens* sequence (Figure S13), thus is a possible dehalogenase. The gene sequence was deposited with GenBank (accession number OP966820). When the amino acid sequence of the putative dehalogenase from strain B was compared with those of the known fluoroacetate dehalogenases, there was a low similarity (approx. 30%) using the BLASTp algorithm. However, when the sequences were aligned using the Constraint-based Multiple Alignment Tool (COBALT), a more complicated picture emerged (Figure 8). Jitsumori *et al.*(42) investigated the mechanism of fluoroacetate dehalogenase from *Burkholderia* sp. FA1 using X-ray crystallography and mutagenesis, which identified key active site residues. A catalytic triad of Asp104, His271, and Asp128 was identified in this enzyme, and alignment with the protein from strain B indicated the presence of a similar triad, but with serine replacing the Asp104, which is the nucleophile in the FA1 enzyme that attacks the fluoromethyl of the substrate. Trp150 of the FA1 enzyme was shown to be essential for the dehalogenation of fluoroacetate,^42^ and the alignment shows the presence of this residue in the strain B protein (position 142). Thus, based on our experimental observations and sequence analysis, we propose that the enzyme in strain B is a new variant of fluoroacetate dehalogenase, or a hydrolase that has a substrate specificity that includes fluorinated compounds. However, it is unlikely that serine acts as the nucleophile in the dehalogenation reaction as a stable ether would be formed after attack on the fluorinated substrate. Beloqui *et al.*(43) identified a hybrid esterase–haloacid dehalogenase, REBr, that also has the catalytic triad of Asp/His/Ser. The serine residue is the nucleophile when the enzyme functions as an esterase, but in the dehalogenation reaction an active site glutamate residue acts as the nucleophile. It is possible that the enzyme in strain B, which otherwise has a low homology to REBr (21.7%), employs another Asp or Glu residue to catalyze defluorination.

### 3.5 Fluorinated Compounds Are Present in Irish Garden Soil

**Figure 9:**
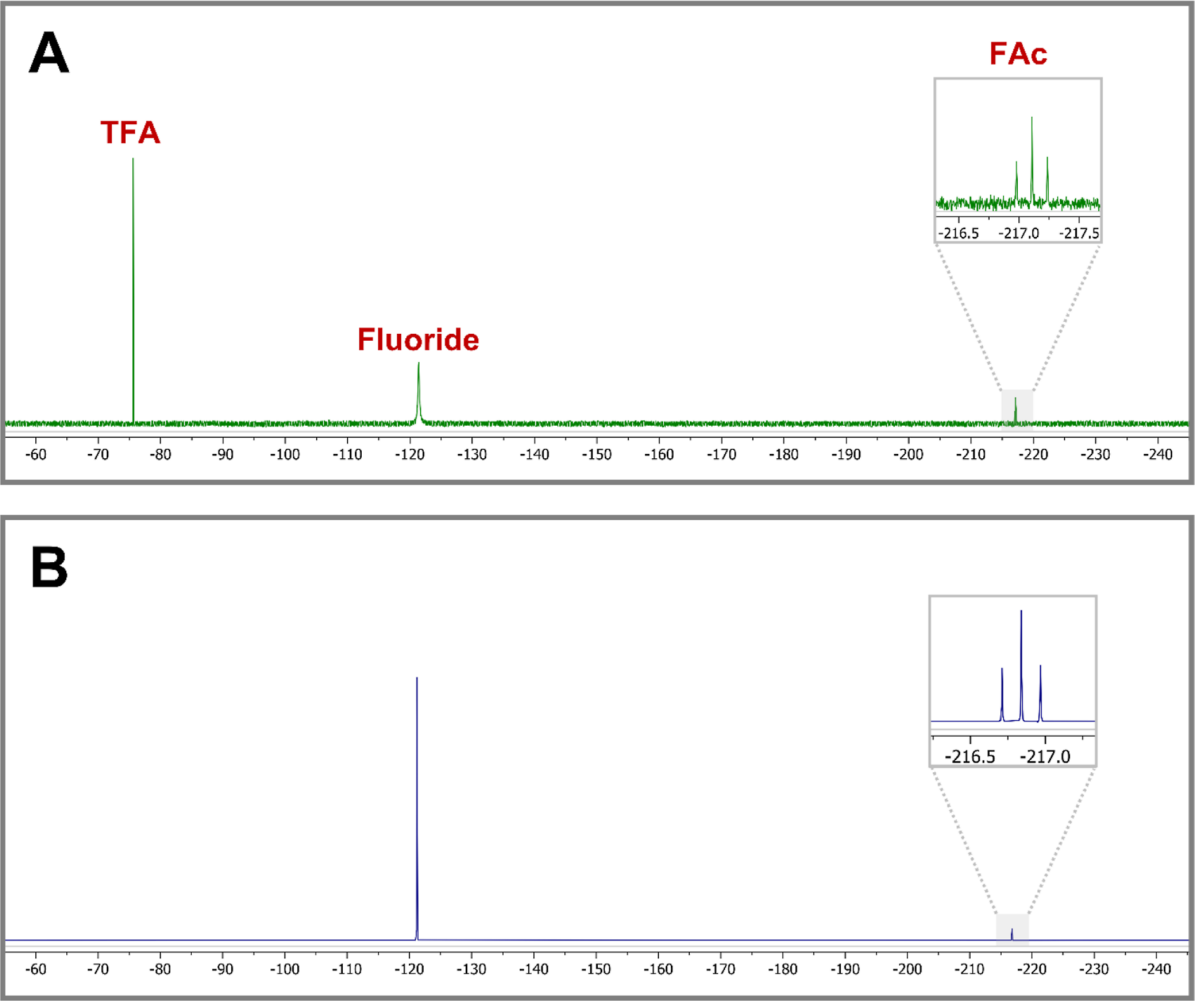
^19^F NMR spectrum of (A) aqueous extract of garden soil and (B) soil microbes after growth in TSB supplemented with NaF.

The detection of defluorinating activity was unexpected in bacteria present in a soil sample taken from an Irish garden. Thus, to investigate if there were any compounds present in the soil that might explain the presence of bacteria capable of enzymatic defluorination, an aqueous extract of approx. 200 g of soil was analyzed by ^19^F NMR. Extreme care was taken not to contaminate the glassware and solvents used. Surprisingly, three resonances were observed that were readily assigned as trifluoroacetate, fluoride ion, and fluoroacetate (Figure 9A) based on their chemical shifts (Figure S14). Two samples of pristine (non-cultivated) soil were also analyzed for fluorinated compounds in the same way, but no resonances were observed by ^19^F NMR, indicating that these compounds are particular to the source of soil. While the presence of trifluoroacetate might be explained by the use of fluorinated pesticides that were degraded in the soil,^44^ the detection of fluoroacetate could not be similarly accounted for. This compound is used as a rodenticide in some countries, but it is not sold in Ireland, which points to a natural source for the compound in the soil sample. Plants that are fluoroacetate producers are found in tropical and sub-tropical regions,^45^ so it is unlikely that this is the origin of the compound. A small number of microorganisms are also known to produce fluoroacetate, most notably *Streptomyces cattleya*, a microorganism isolated from soil^46^ and from which the first fluorinase was identified,^47^ and more recently *Streptomyces* MA37.^48^ Thus, it is most likely that the source of fluoroacetate is bacterial. To investigate this, the SM was cultivated in TSB supplemented with 10 mM sodium fluoride. After 7 days, the culture supernatant was freeze dried, redissolved in D_2_O, and analyzed by ^19^F NMR. In addition to fluoride ions, there was another resonance at approx. −217 ppm and which had the characteristic triplet of a monofluoromethyl (Figure 9B), indicating that fluoroacetate, or a very closely related compound, was biosynthesized by a bacterium present in the consortium. The same experiment was conducted with consortia grown from the two uncultivated soils, but no organic fluorine was detected (Figure S15). Thus, there are microorganisms in the garden soil that can produce and degrade fluoroacetate, which are not present in the uncultivated soil. It is likely that these species were introduced from plants and compost acquired from nurseries, which have subsequently become established in the microbial community; furthermore, the use of fluorinated herbicides may have contributed to the enrichment of such organisms.

### 3.6 Implications

Our understanding of the environmental fate of the myriad-fluorinated compounds that are in use today is sparce, and this has led to poor decision making relating to their regulation. It is important to have a clear understanding of the likely fate of new fluorinated compounds before they reach the market. The compounds used in this study are not currently used in any application, but fluorinated SAPs are of broad interest as biomaterials. Under the conditions that were employed, the biodegradability of the peptides and their constituent amino acids was confirmed, which is encouraging and suggests that such materials will not present an environmental risk. The biodegradation experiments with soil microorganisms led to the serendipitous discovery of the enzymatic defluorination of MfeGly, which was previously unknown. Our experiments indicate that the activity is related to the known fluoroacetate dehalogenase, but that the enzyme responsible seems to be a new variant. The presence of such an enzyme in a garden soil from Ireland was initially puzzling, but the detection of organofluorine compounds in soil extracts provided a possible explanation as to the origins of the defluorinating enzyme. Therefore, in other environments where fluoroacetate or similar fluorinated compounds are present, new dehalogenating enzymes might have evolved, and these might be subsequently applied to the remediation of organofluorine-polluted ecosystems. Finally, the presence of bacteria capable of biosynthesizing fluoroacetate indicates that the highly unusual fluorinases that catalyze carbon–fluorine bond formation can be found in unexpected microbial communities, and that well-cultivated gardens can be a source of these biotechnologically important enzymes. The focus is now on identifying the bacterium/bacteria that produce fluoroacetate and further investigating the hydrolase enzyme responsible for fluoroacetate degradation.

## Supporting Information Available

Details of peptide synthesis with associated analytical data; additional ^19^F NMR spectra of peptide biotransformation by soil bacteria; mass spectral data for homoserine; phylogram showing homology of isolated strain with other *Serratia* spp.; gene sequence alignment; ^19^F NMR spectra of pristine soil extracts; and HPLC chromatograms of peptide control experiments (PDF)

## Abbreviations

MfeGly: monofluoroethylglycine
DfeGly: difluoroethylglycine
TfeGly: trifluoroethylglycine
