# Supplementary material for: Biodegradation of Amphipathic Fluorinated Peptides Reveals a New Bacterial Defluorinating Activity and a New Source of Natural Organofluorine Compounds
Source: Environ Sci Technol. 2023 Jun 21;57(26):9762–72. doi: 10.1021/acs.est.3c01240 (PMC10324308; doi:10.1021/acs.est.3c01240)
Supplement: Supplementary file 1 — es3c01240_si_001.pdf [file es3c01240_si_001.pdf]

Biodegradation of amphipathic fluorinated peptides reveals a new bacterial defluorinating activity and a new source of natural organofluorine compounds

Mohd Faheem Khan [a], Suvrat Chowdhary [b], Beate Koksch [b] and Cormac D. Murphy [a*]*

[a] School of Biomolecular and Biomedical Science, University College Dublin, Belfield, Dublin 4, Ireland

[b] Institute of Chemistry and Biochemistry, Freie Universität Berlin, Arnimallee 20, 14195 Berlin, Germany

Supplemental Information

Summary: 17 pages, 2 pages of text, 15 figures.

Contents

	Page no.
Synthesis of fluorinated amino acids MfeGly, DfeGly and TfeGly	S3
Figure S1. ^{1}H NMR spectrum of H-MfeGly-OH	S5
Figure S2. ^{19}F NMR spectrum of H-MfeGly-OH	S5
Figure S3. ^{1}H NMR spectrum of H-DfeGly-OH	S6
Figure S4. ^{19}F NMR spectrum of H-DfeGly-OH	S6
Figure S5. ^{1}H NMR spectrum of H-TfeGly-OH	S7
Figure S6. ^{19}F NMR spectrum of H-TfeGly-OH	S7
Figure S7. Chemical structures of peptides Abuk16, MfeGlyK16, DfeGlyK16 and TfeGlyK16	S8
Figure S8. HPLC chromatograms (DAD-280nm) of a) AbuK16, b) MfeGlyK16, c) DfeGlyK16 and d) TfeGlyK16 (0.5 mM) dissolved in sole buffer without enzyme and recorded after 0 h (top) and 48 h (bottom) incubation (37 °C). Aliquots were further diluted with the quenching solution, accordingly. Chromatograms are normalized to the reference Ac-[4]Abz-Gly-OH (t_R: 12.5 min).	S9
Figure S9. MS detection of dipeptide fragments Abu-Lys, MfeGly-Lys, DfeGly-Lys, and TfeGly-Lys (isolated from the highlighted HPLC signal) after incubation of AbuK16, MfeGlyK16, DfeGlyK16 and TfeGlyK16 (black chromatograms) with the serine protease β-trypsin for 24 h (colored HPLC chromatograms).	S10
Figure S10. ^{19}F NMR spectra after fluorinated peptides were incubated with the soil microbial consortium.	S11
Figure S11. Mass spectra of silylated MfeGly (A) and homoserine (B).	S12
Figure S12. Phylogram illustrating the homology of the 16S rRNA gene sequence of strain B with *Serratia* spp.	S13
Figure S13. Sequence alignment of the putative fluoroacetate dehalogenase from *S. marescens* and the gene identified in strain B.	S14
Figure S14. Comparison of the chemicals shifts of resonances detected from aqueous soil extracts with standards of trifluoroacetate, fluoride ion and fluoroacetate.	S15
Figure S15. ^{19}F NMR spectra of soil microbial consortia (SM1 and 2) grown from two uncultivated soils and incubated with fluoride ion.	S16

Synthesis of fluorinated amino acids MfeGly, DfeGly and TfeGly

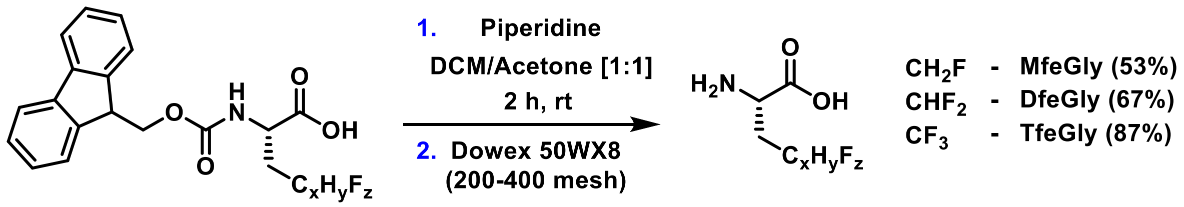

Scheme S1. Synthesis of H-MfeGly-OH, H-DfeGly-OH and H-TfeGly-OH based on their corresponding Fmoc-protected derivatives.

The Fmoc-protected amino acids were synthesised on the gram scale according to recently published protocols by Koksch and co-workers.[1, 2]

As a general procedure, the Fmoc-protected amino acid MfeGly (500 mg, 1.45 mmol, 1 equiv.), DfeGly (500 mg, 1.38 mmol, 1 equiv.) or TfeGly (500 mg, 1.33 mmol, 1 equiv.) was dissolved in a DCM/acetone mixture [10 mL] containing 2% piperidine for Fmoc-deprotection. This reaction mixture was stirred for 2 h. Afterwards, the solution was evaporated, and the crude leftover dried *in vacuo* overnight. The crude product was subsequently dissolved in MilliQ water [10 mL] and washed with EtOAc (3*5 mL) and $CHCl_3$ (3*5 mL) to remove Fmoc-derived impurities. Upon that ion-exchange beads (Dowex 50WX8, 200-400 mesh, 3.5 g) were added to the aqueous phase and the reaction mixture was stirred slowly for 2 hours. Thereafter the resin was packed into a flash column and washed with water [50 mL]. The L-amino acid were finally eluted from the resin with a 1M NH_3 [20 mL] and subsequently dried by lyophilization.

The title compounds H-MfeGly-OH (93.9 mg, 0.77 mmol, 53%), H-DfeGly-OH (129.7 mg, 0.93 mmol, 67%) and H-TfeGly-OH (183.4 mg, 1.16 mmol, 87%) were obtained as white powders.

H-MfeGly-OH ($C_4H_8FNO_2$)

^{1}H NMR (600 MHz, D_2O) δ = 4.60 – 4.46 (m, 2H), 3.75 (s, 1H), 2.27 – 2.05 (m, 2H).

^{19}F NMR (565 MHz, D_2O) δ = -219.04 – -219.41 (m, 1F).

H-DfeGly-OH ($C_4H_7F_2NO_2$)

^{1}H NMR (600 MHz, D_2O) δ = 6.06 – 5.89 (m, 1H), 3.95 – 3.69 (m, 1H), 2.51 – 2.20 (m, 2H).

^{19}F NMR (565 MHz, D_2O) δ = -115.18 – -117.83 (m, 2F).

H-TfeGly-OH ($C_4H_6F_3NO_2$)

^{1}H NMR (600 MHz, D_2O) δ = 3.76 – 3.48 (m, 1H), 2.79 – 2.59 (m, 1H), 2.53 – 2.26 (m, 1H).

^{19}F NMR (565 MHz, D_2O) δ = -63.51 – -63.60 (m, 3F).

Figure S1. ^{1}H NMR spectrum of H-MfeGly-OH

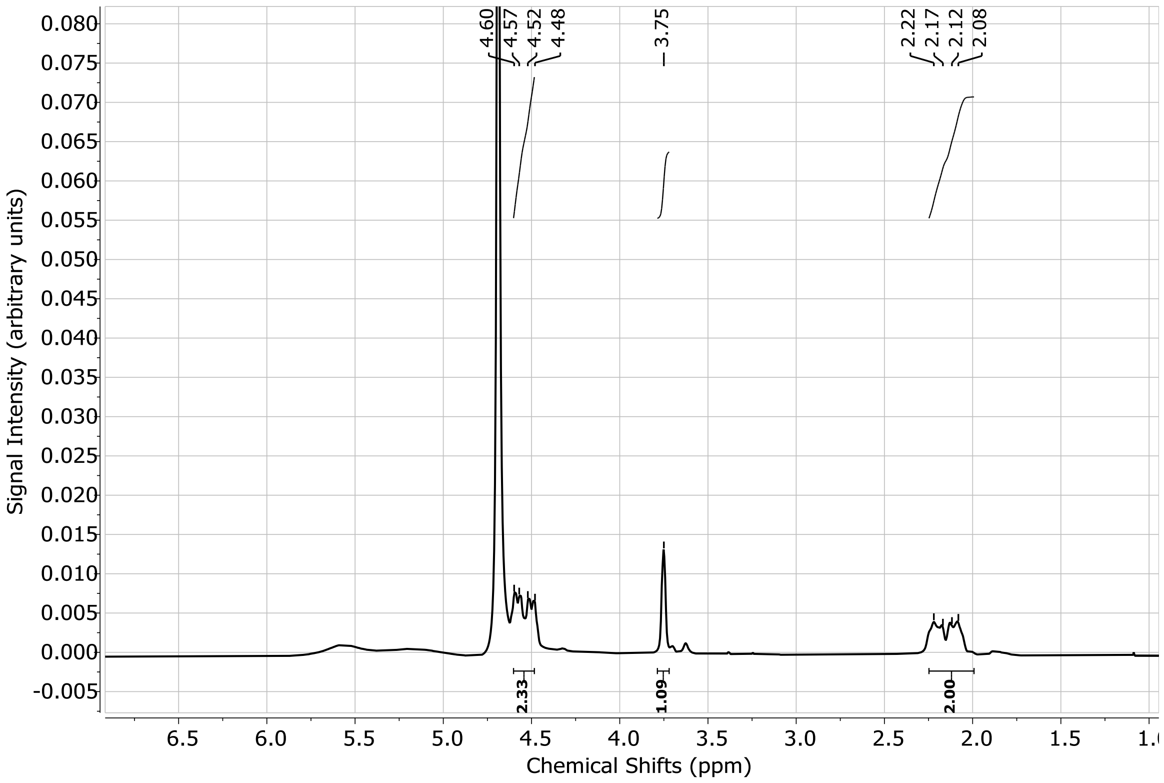

Figure S2. ^{19}F NMR spectrum of H-MfeGly-OH

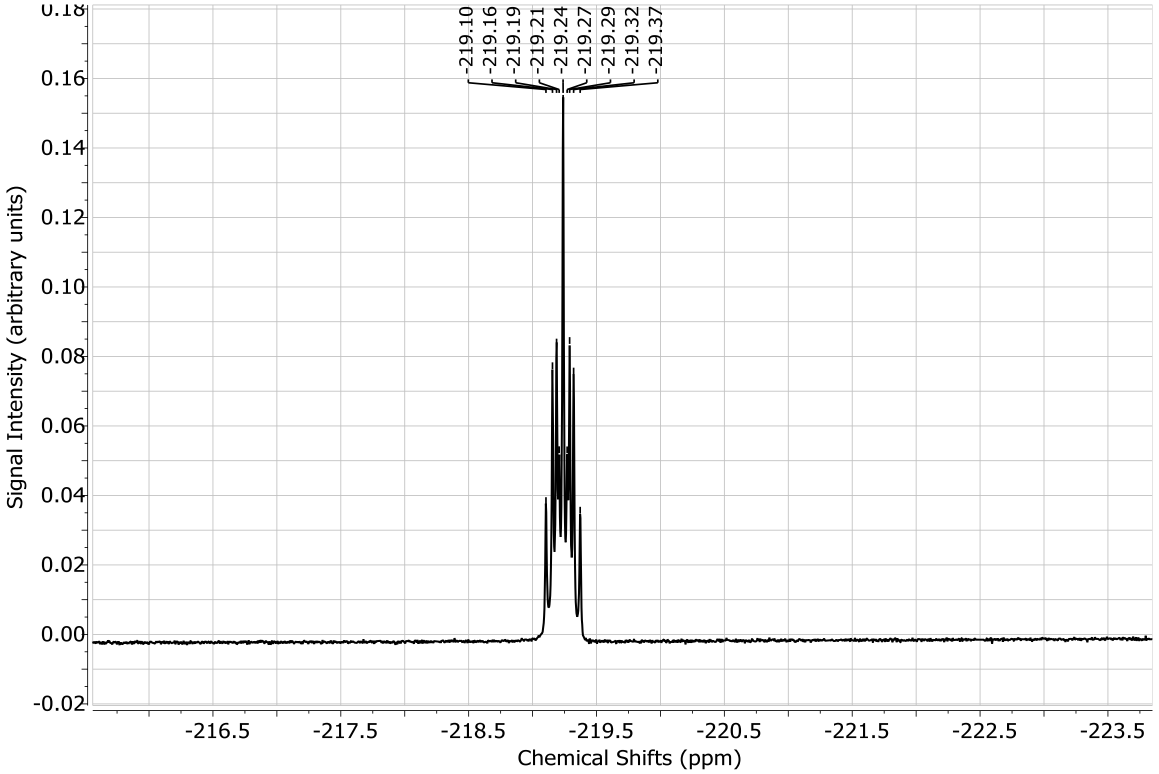

Figure S3. ^{1}H NMR spectrum of H-DfeGly-OH

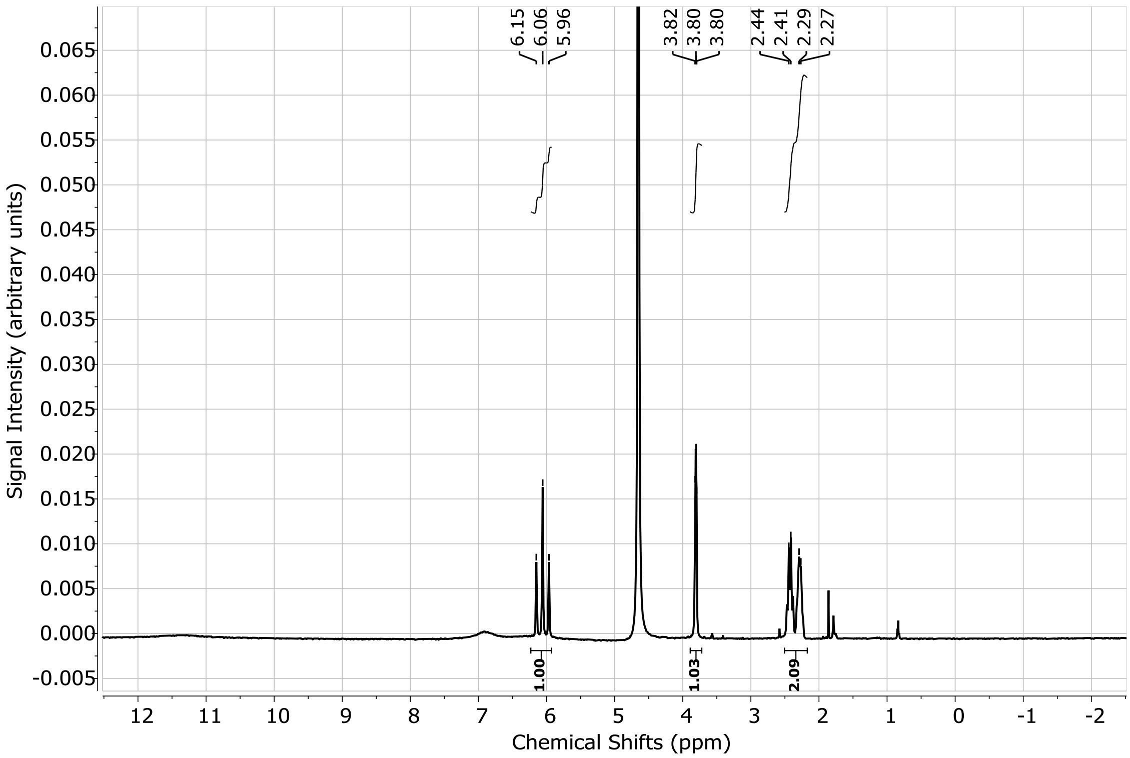

Figure S4. ^{19}F NMR spectrum of H-DfeGly-OH

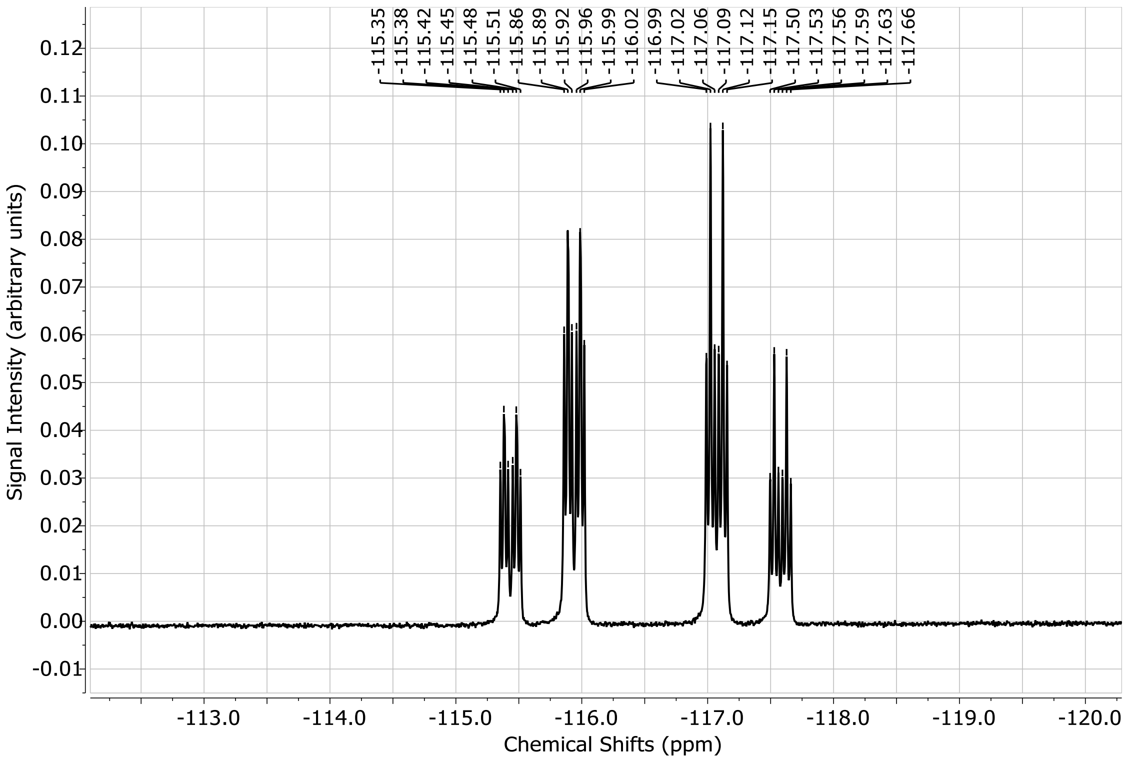

Figure S5. ^{1}H NMR spectrum of H-TfeGly-OH

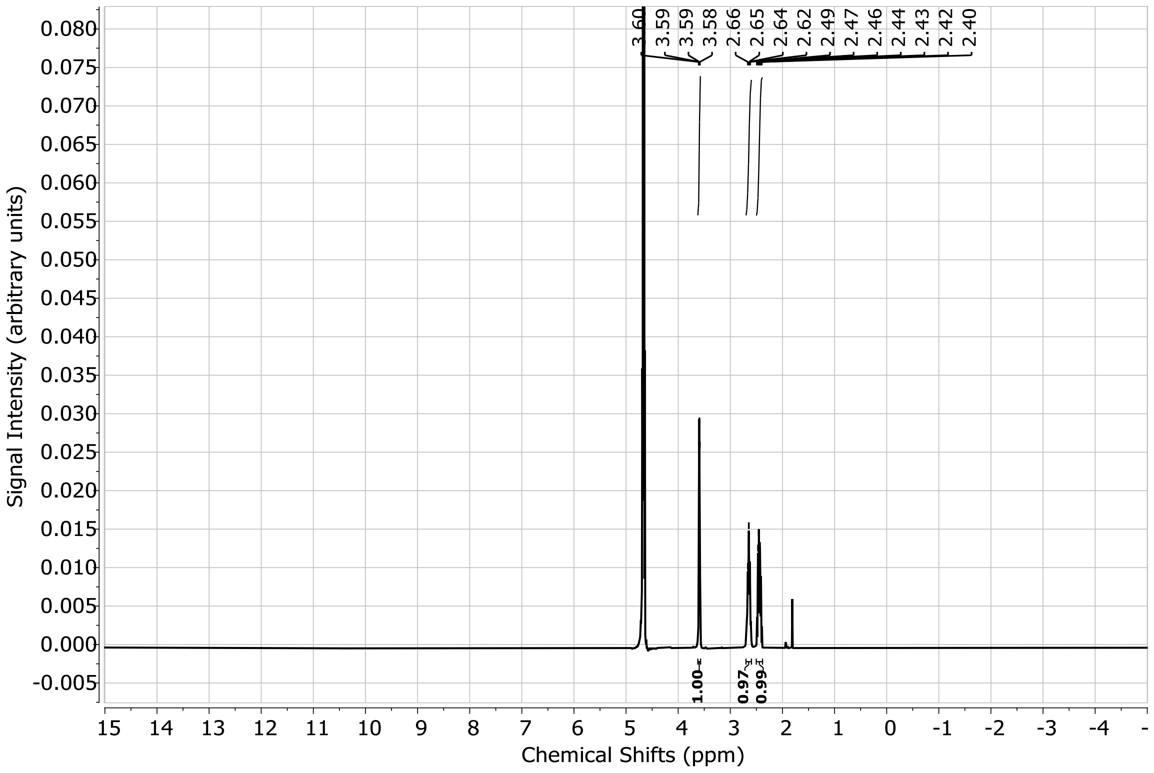

Figure S6. ^{19}F NMR spectrum of H-TfeGly-OH

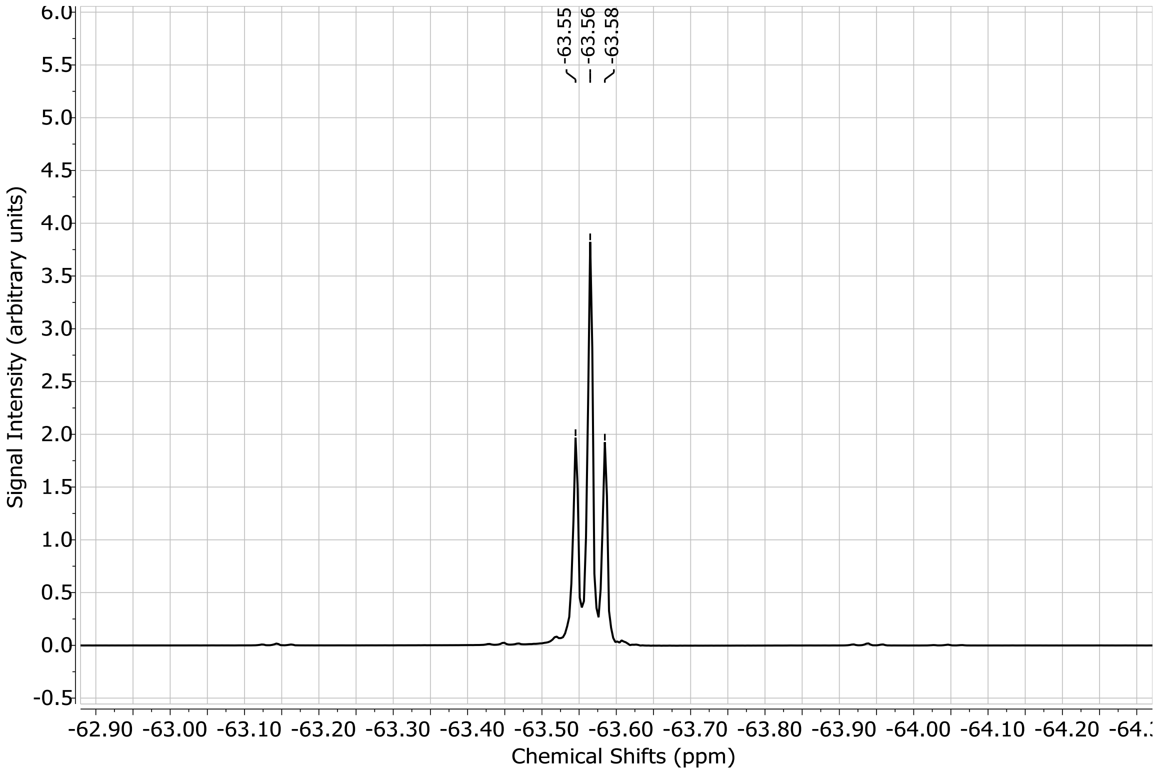

Figure S7. Chemical structures of peptides Abuk16, MfeGlyK16, DfeGlyK16 and TfeGlyK16

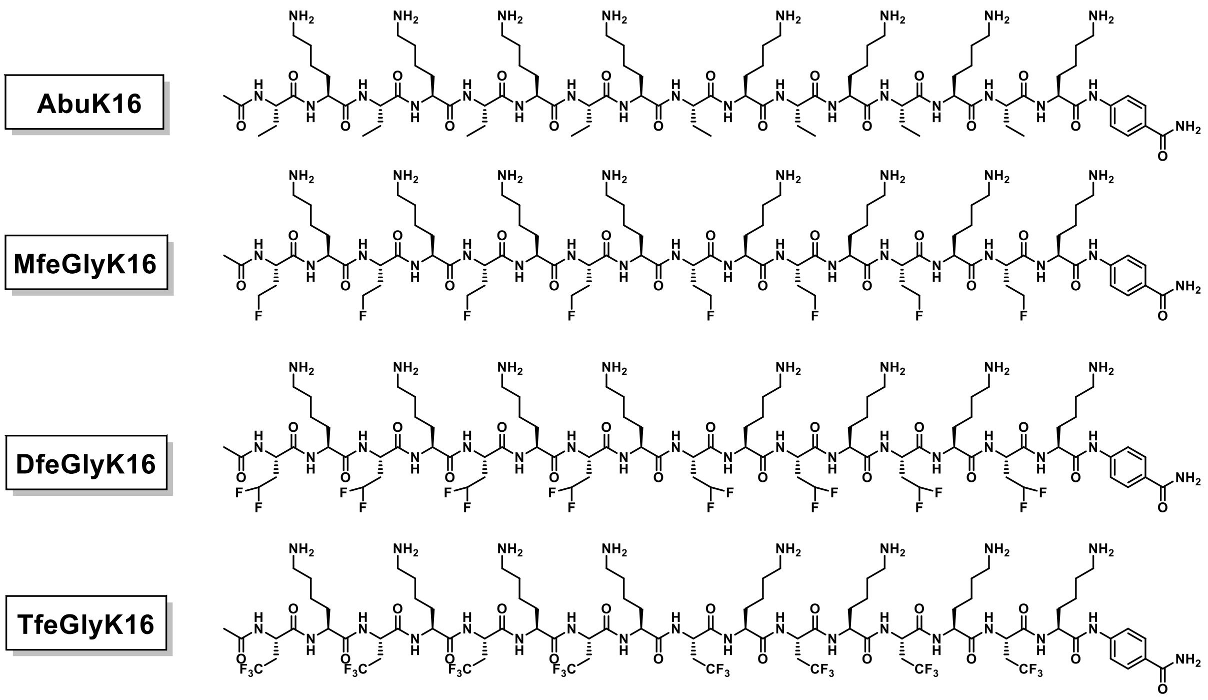

Figure S8. HPLC chromatograms (DAD-280nm) of a) AbuK16, b) MfeGlyK16, c) DfeGlyK16 and d) TfeGlyK16 (0.5 mM) dissolved in sole buffer without enzyme and recorded after 0 h (top) and 48 h (bottom) incubation (37 °C). Aliquots were further diluted with the quenching solution, accordingly. Chromatograms are normalized to the reference Ac-[4]Abz-Gly-OH (t_R: 12.5 min).

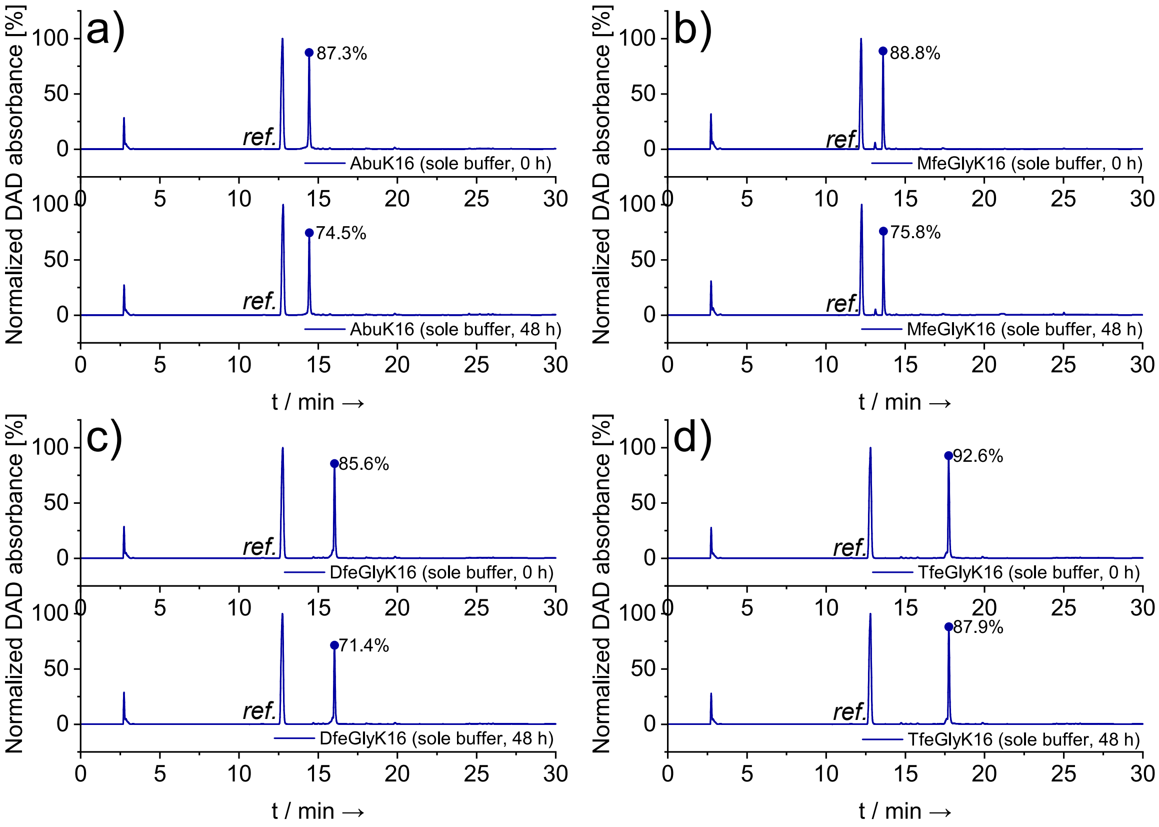

Figure S9. MS detection of dipeptide fragments Abu-Lys, MfeGly-Lys, DfeGly-Lys, and TfeGly-Lys (isolated from the highlighted HPLC signal) after incubation of AbuK16, MfeGlyK16, DfeGlyK16 and TfeGlyK16 (black chromatograms) with the serine protease β-trypsin for 24 h (colored HPLC chromatograms).

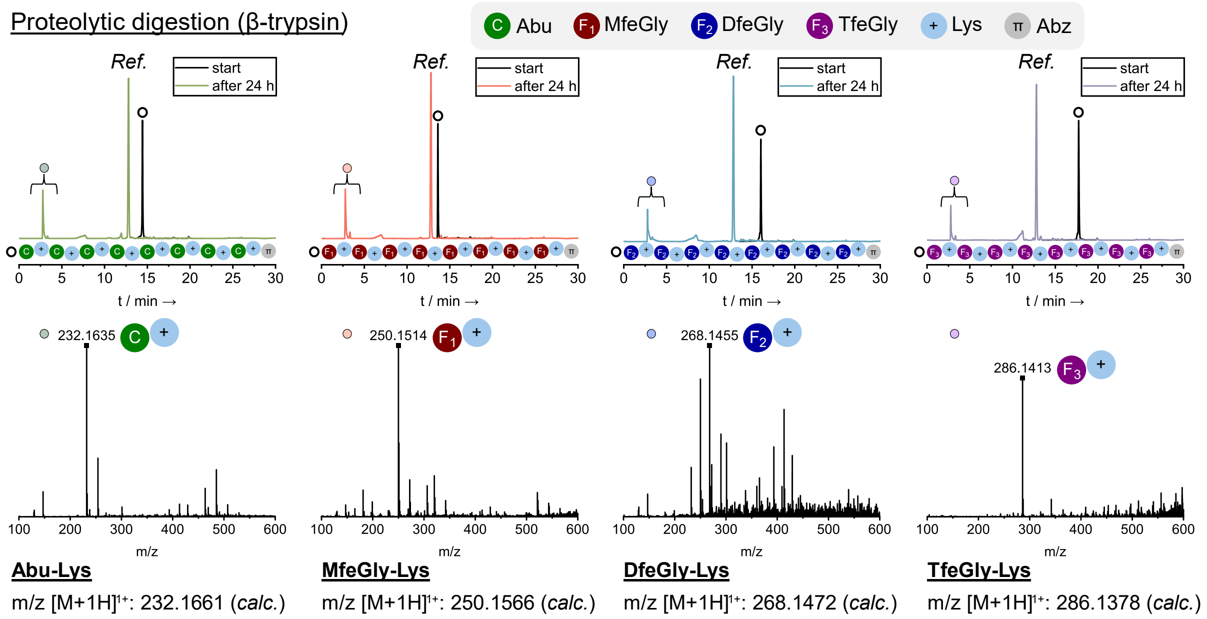

Figure S10. ^{19}F NMR spectra after fluorinated peptides were incubated with the soil microbial consortium. Black spectra are from control experiments in which the peptides were incubated with TSB for 48 h.

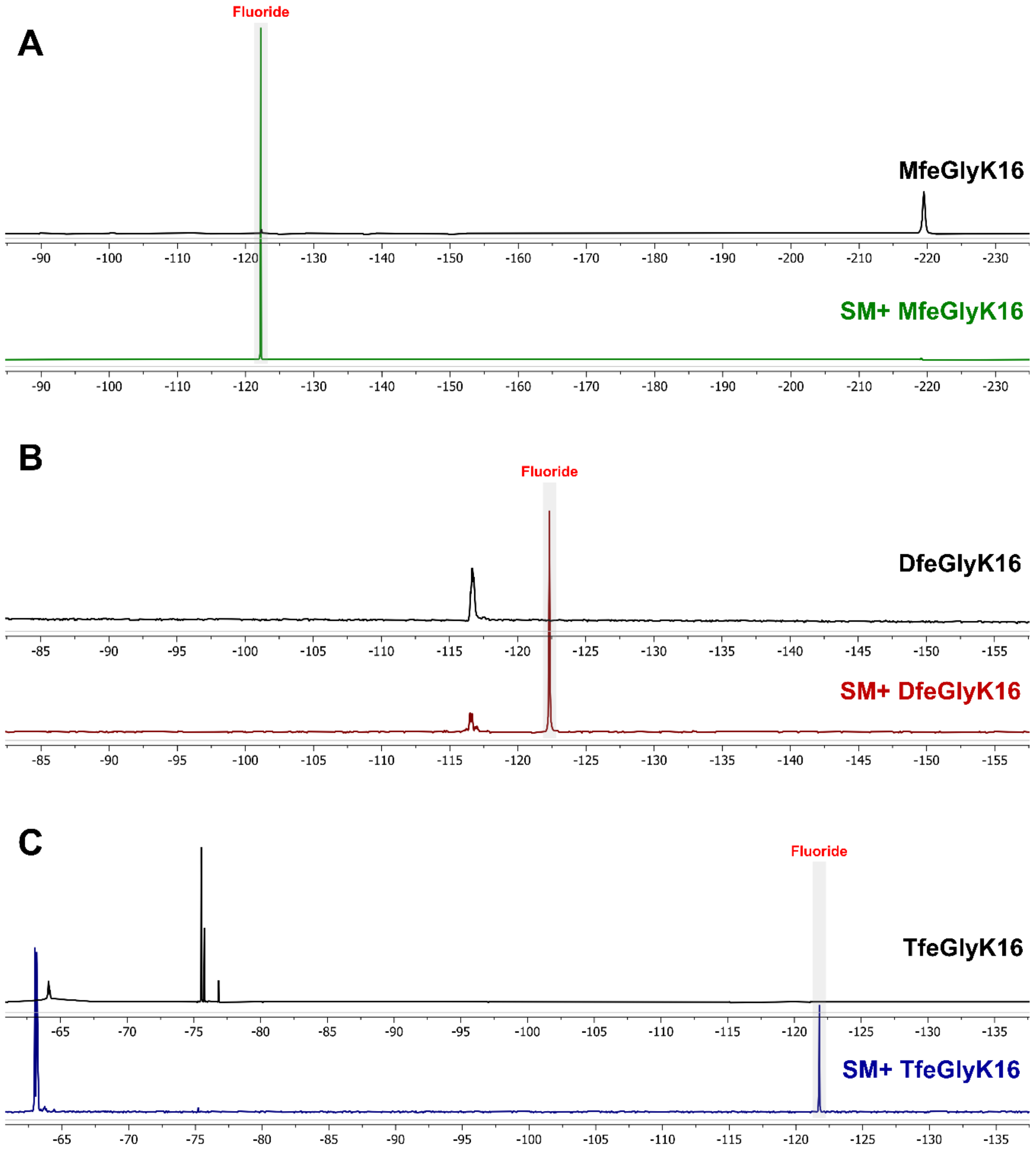

Figure S11. Mass spectra of silylated MfeGly (A) and homoserine (B).

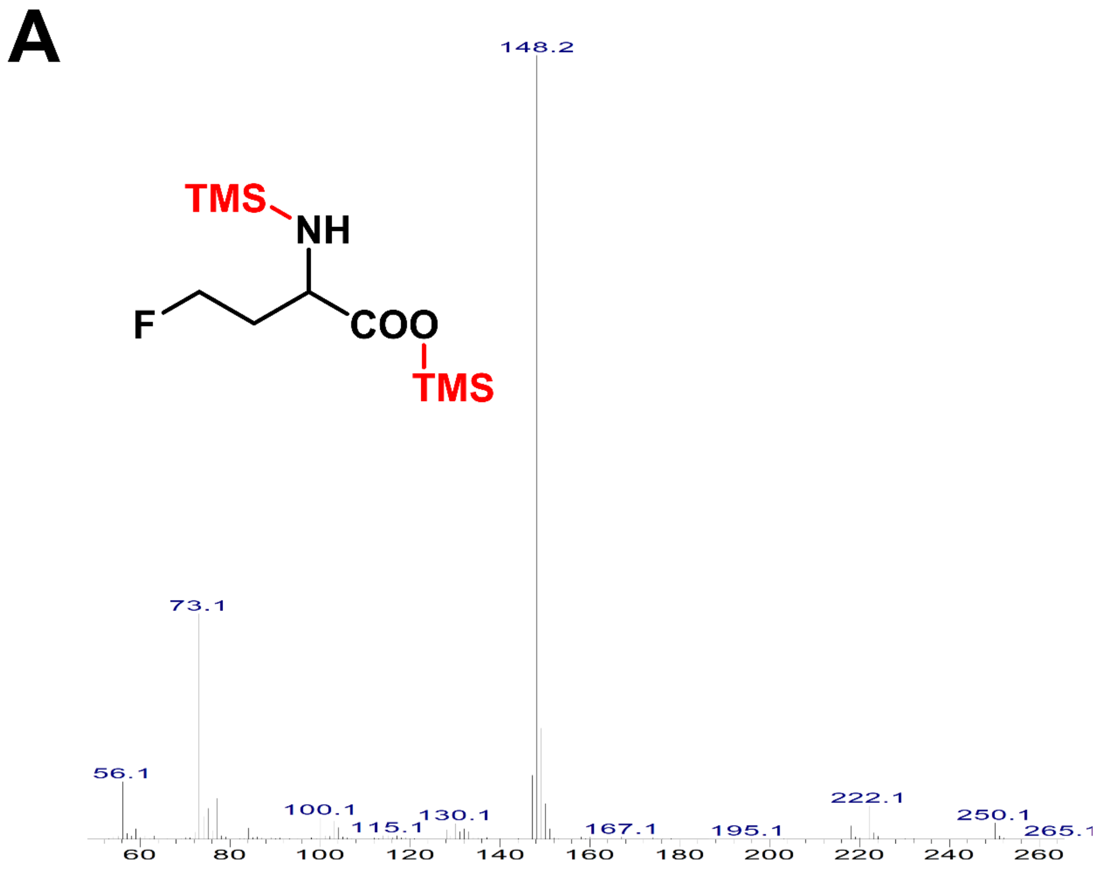

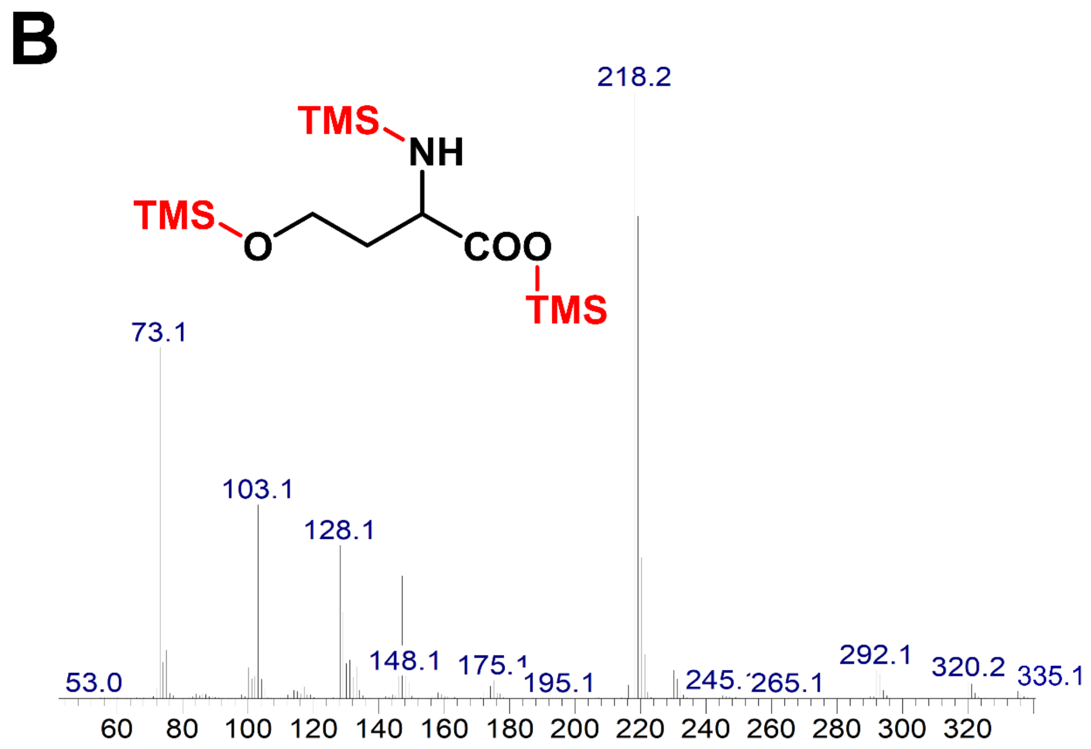

Figure S12. Phylogram illustrating the homology of the 16S rRNA gene sequence of strain B with *Serratia* spp.

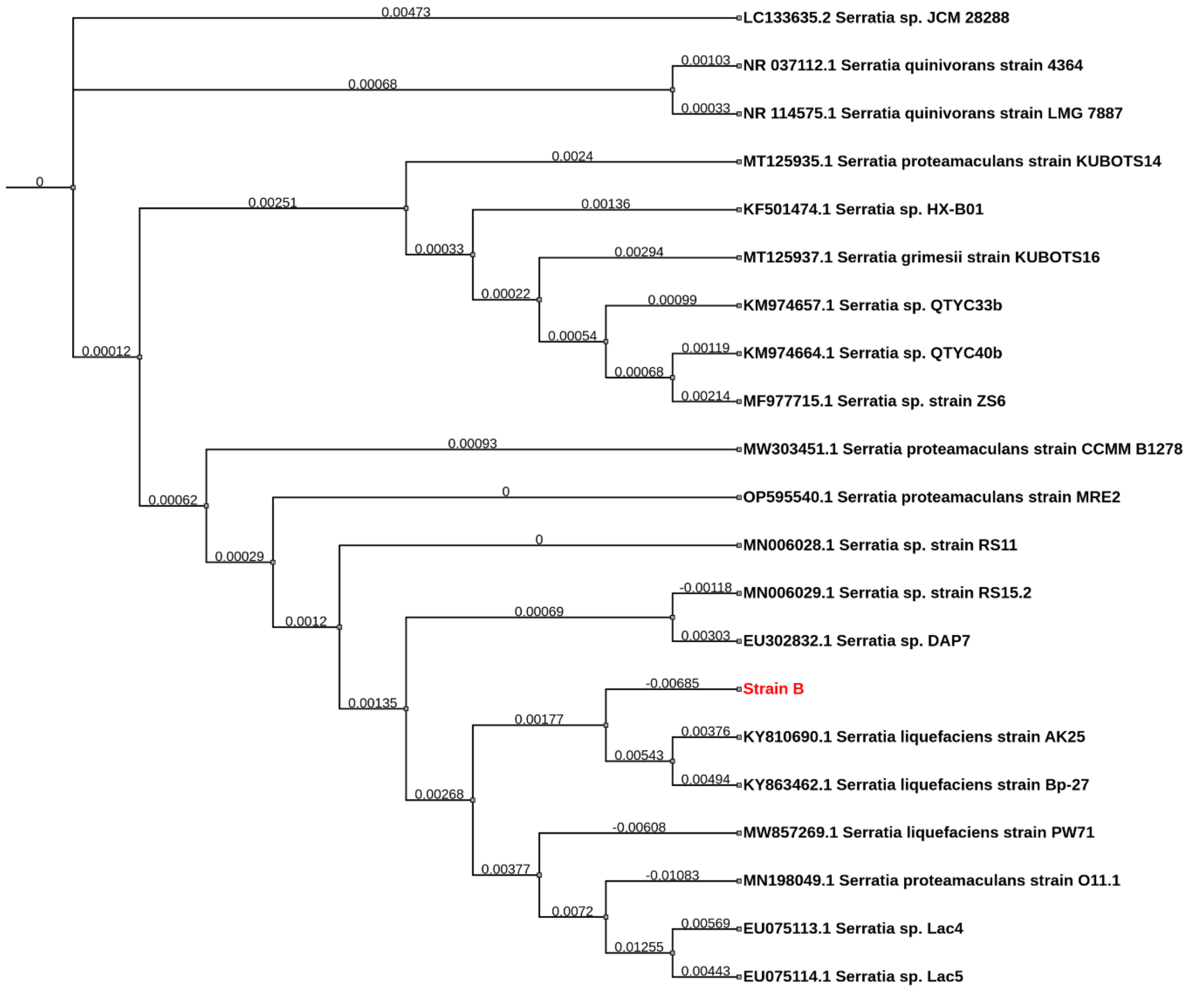

Figure S13. Sequence alignment of the putative fluoroacetate dehalogenase from *S. marescens* and the gene identified in strain B.

```
Query: CVE64293.1 Fluoroacetate dehalogenase [Serratia marcescens] Query ID: lcl|Query_58143 Length: 259


>NODE_12_length_204635_cov_6.825444
Sequence ID: Query_58156 Length: 204635
Range 1: 91080 to 91871

Score:385 bits(990), Expect:7e-124,
Method:Compositional matrix adjust.,
Identities:215/264(81%), Positives:232/264(87%), Gaps:5/264(1%)

Query  1      MSTFLYGAHVHANGIRQHYLRYGGHGPALILIPGITSPAITWGFVAERLGEKYDVYVLDV  60
              MS FLYGA+V ANGIRQHYLRYGG GP +ILIPGITSPAITWGFVAERL EKYD YVLDV
Sbjct  91080  MSHFLYGANVQANGIRQHYLRYGGQGPVVILIPGITSPAITWGFVAERLAEKYDTYVLDV  91259

Query  61     RGRGLSSSGPELAYDaet-----CAHAAALNLASYAllGHSMGARFALRAAVLQPAGVRR  115
              RGRGLS+SGP+LAYDAET      + A AL L +YALLGHSMGARFALRAA L PAGVRR
Sbjct  91260  RGRGLSASGPDLAYDAETCAQDITSFACALQLENYALLGHSMGARFALRAAALHPAGVRR  91439

Query  116    LVLVDPPVSGPGRRAYPGQWPWYADSIRQSLPGMSAEQMRAFCPTWSEEQRRLRAEWLHT  175
              LVL+DPPVSGPGRR YPG+WPWY DSIRQSL GM+AEQMR +CP WSE QR+LRAEWLHT
Sbjct  91440  LVLIDPPVSGPGRREYPGKWPWYVDSIRQSLLGMNAEQMRTYCPNWSESQRQLRAEWLHT  91619

Query  176    CYEPAIQRAYDDFHQVDSHRDYPLLTLPTllIAAGKGGVIQPEDRAEIRALQPEIAIVEV  235
              CYEPAIQRAY+DFHQVDSHRDYP L++PTLL+ AG GGVIQ ED AEIRALQPEI +  V
Sbjct  91620  CYEPAIQRAYEDFHQVDSHRDYPALSMPTLLMVAGLGGVIQQEDEAEIRALQPEITLAHV  91799

Query  236    ENAGHMIPWDDFDGFFRALGDFLD  259
              ENAGHMIPWDDFDGFFRALG+FLD
Sbjct  91800  ENAGHMIPWDDFDGFFRALGNFLD  91871
```

Figure S14. Comparison of the chemicals shifts of resonances detected from aqueous soil extracts with standards of trifluoroacetate, fluoride ion and fluoroacetate.

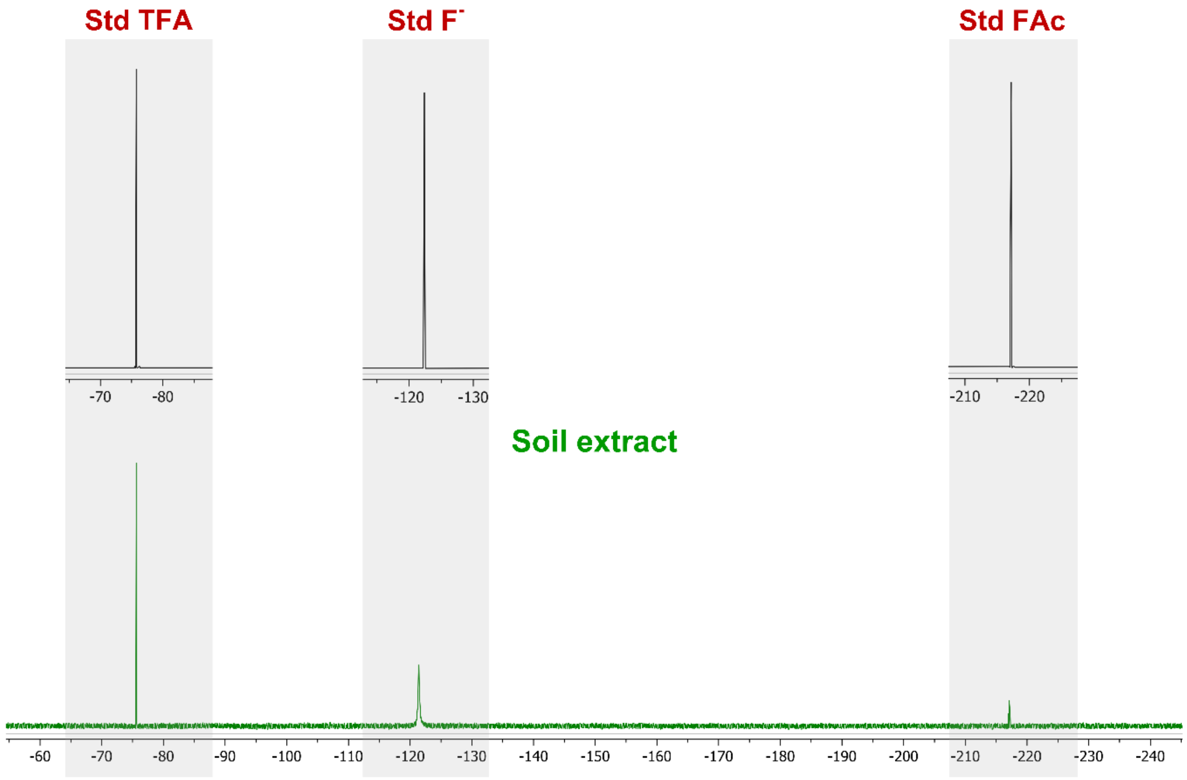

Figure S15. ^{19}F NMR spectra of soil microbial consortia (SM1 and 2) grown from two uncultivated soils and incubated with fluoride ion.

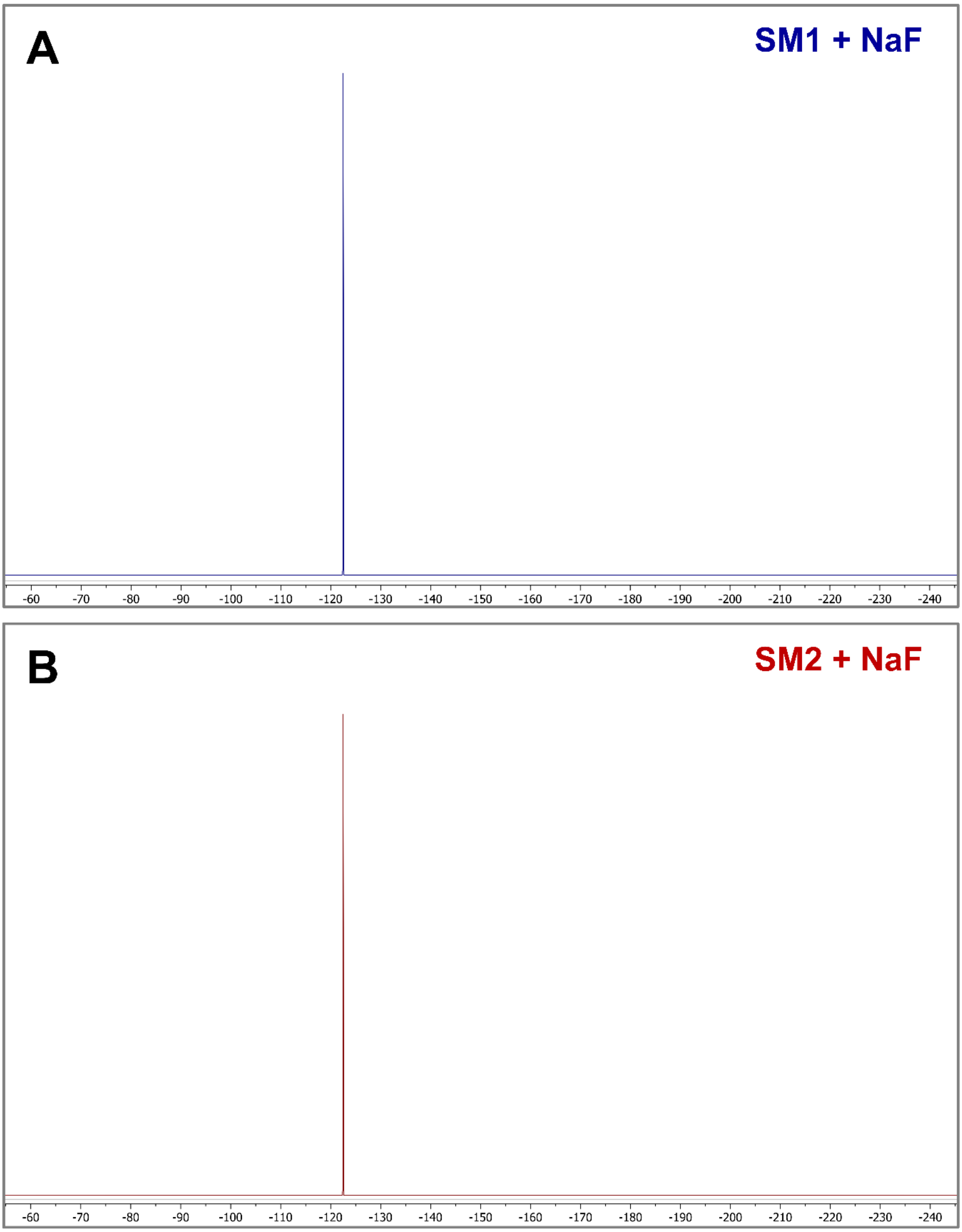

Notes and References

1. T. Hohmann, M. Dyrks, S. Chowdhary, M. Weber, D. Nguyen, J. Moschner and B. Koksch, *The Journal of Organic Chemistry*, 2022, **87**, 10592-10604.
2. S. Chowdhary, R. F. Schmidt, A. K. Sahoo, T. tom Dieck, T. Hohmann, B. Schade, K. Brademann-Jock, A. F. Thünemann, R. R. Netz, M. Gradzielski and B. Koksch, *Nanoscale*, 2022, **14**, 10176-10189.
